# Proteomic analysis and candidate allergenic proteins in *Populus deltoides* CL. “2KEN8” mature pollen

**DOI:** 10.3389/fpls.2015.00548

**Published:** 2015-07-29

**Authors:** Jin Zhang, Li-Shuan Wu, Wei Fan, Xiao-Ling Zhang, Hui-Xia Jia, Yu Li, Ya-Fang Yin, Jian-Jun Hu, Meng-Zhu Lu

**Affiliations:** ^1^State Key Laboratory of Tree Genetics and Breeding, Key Laboratory of Tree Breeding and Cultivation of the State Forestry Administration, Research Institute of Forestry, Chinese Academy of ForestryBeijing, China; ^2^Co-Innovation Center for Sustainable Forestry in Southern China, Nanjing Forestry UniversityNanjing, China; ^3^Research Institute of Wood Industry, Chinese Academy of ForestryBeijing, China; ^4^Key Laboratory of Photobiology, Institute of Botany, Chinese Academy of Sciences, Beijing, China, University of Chinese Academy of SciencesBeijing, China

**Keywords:** allergen, MALDI-TOF/TOF MS/MS, pollen, *Populus deltoids*, profilin, proteomics, two-dimensional gel electrophoresis

## Abstract

Proteomic analysis was used to generate a map of *Populus deltoides* CL. “2KEN8” mature pollen proteins. By applying 2-D electrophoresis, we resolved 403 protein spots from mature pollen. Using the matrix-assisted laser desorption/ionization time time-of-flight/time-of-flight tandem mass spectrometry method, we identified 178 distinct proteins from 218 protein spots expressed in mature pollen. Moreover, out of these, 28 proteins were identified as putative allergens. The expression patterns of these putative allergen genes indicate that several of these genes are highly expressed in pollen. In addition, the members of profilin allergen family were analyzed and their expression patterns were compared with their homologous genes in *Arabidopsis* and rice. Knowledge of these identified allergens has the potential to improve specific diagnosis and allergen immunotherapy treatment for patients with poplar pollen allergy.

## Introduction

In spermatophytes, pollen grains are the dispersal agents of sperm cells and are vital for successful sexual reproduction and subsequent seed and fruit production (Sheoran et al., 2007). The development of pollen, microsporogenesis and microgametogenesis involves the coordinated expression of several genes in different tissues of an anther (Mccormick, 2004; Ma, 2005), and pollen grains at maturity contain a large number of transcripts with designated roles in cell wall metabolism, cytoskeleton formation, cell signaling, and vesicle transport (Becker et al., 2003; Honys and Twell, 2003; Pina et al., 2005). It has been reported that pollens are a major cause of Type I allergies due to the presence of several allergens (Nakamura and Teshima, 2013). Manifestation of allergic diseases, spanning from mild rhinitis to anaphylaxis is a major health problem, affecting the quality of life of millions of people all over the world (Sircar et al., 2012). More than 30% of the world population is affected by different kinds of allergy, caused by naturally occurring as well as synthetically produced compounds and their prevalence is increasing daily (Singh and Shahi, 2008).

The proteome is the entire set of proteins expressed by a genome, cell, tissue, or organism (Wilkins et al., 1996). It is highly dynamic and depends on cell cycle, environmental influences and tissue/cell type. Rapid advances in proteomic technologies, along with completion of many plant species genome sequencing projects and availability of comprehensive public sequence databases, have provided tremendous impetus to plant proteomics research (Canovas et al., 2004; Hirano et al., 2004).

In recent years, proteomic techniques that target protein allergens, i.e., allergenomics, emerged as powerful tools for comprehensive allergen analysis (Akagawa et al., 2007; Picariello et al., 2011). At first, proteomic analyses were used to detect novel allergens by identifying proteins following separation by 2-DE and MS (Nakamura and Teshima, 2013). Compared to conventional methods based upon protein isolation processes, proteomics has accelerated identification of numerous allergens in plants. Furthermore, novel allergenomics techniques, which consider the properties (biochemical, structural, reactivity) of the allergens, have been developed (Kitta et al., 2006; Yano and Kuroda, 2008; Shahali et al., 2012). However, up to recent times, very little was known about the molecular basis of poplar pollen allergy, one of the more common causes of allergy symptoms, particularly in spring.

On this basis, the objective of the present study was to identify likely allergenic proteins of *Populus* pollen. The genus *Populus* contains approximately 30 species of woody plants, all found in the Northern hemisphere and exhibiting some of the fastest growth rates observed for trees growing in temperate climate (Taylor, 2002). *P. deltoides* is a poplar species with a high yield, fine wood quality, strong adaptability and disease resistance. Therefore, it is used widely worldwide as an important woody species. However, *P. deltoides* releases large amounts of pollens in spring and these pollens are surmised to cause the allergenic response in human. Such pollens belong to the most important elicitors of allergy in adults and adolescents (Vieths et al., 2002). Allergy is an adverse reaction to normally harmless substances, such as allergens, by the immune system, and it involves immune response mediated by an increased amount of immunoglobulin E (IgE) or IgG antibodies (Bohle, 2004). Once sensitized, human can become allergic to homologous proteins of other pollens or present in food, via cross-reactivity (Vieths et al., 2002). The inhalation of pollen from several birch trees and grasses is the main cause of primary sensitization in humans (Bartra et al., 2009).

The majority of allergens present in plants belong to four families: pathogenesis-related protein 10 (PR-10 protein, birch allergen Bet v 1 homologs), thaumatin-like proteins (TLP, PR-5 proteins), non-specific lipid transfer proteins (nsLTPs, PR-14 proteins) and profilins (PRF) (Breiteneder and Ebner, 2000). Due to the common structure and properties of such allergens over a wide range of plant species, genera and even families, allergy cross-reactivity has been frequently observed.

By combining two-dimensional gel electrophoresis (2-DE) with matrix-assisted laser desorption/ionization time time-of-flight/time-of-flight tandem mass spectrometry (MALDI-TOF/TOF MS/MS), and by using the available databases for *P. trichocarpa* and other plant species, a comprehensive analysis of *P. deltoides* CL. “2KEN8” mature pollen proteome was performed in the present work. Many of the proteins identified in this study have predicted roles in defense mechanisms, energy conversion, pollen germination, and pollen tube growth, and possibly in sperm cell formation. To our knowledge, there has been no previous proteomic study to predict the pollen allergens of poplar. Thus, we aimed to identify expressed proteins and the likely allergens in poplar mature pollen.

## Materials and methods

### Plant materials and pollen collection

“2KEN8” trees, one of the widely grown high-yield *P. deltoids* in China were obtained from nursery of Chinese Academy of Forestry, Beijing, China. The flowering branches were cut and then placed in water in a greenhouse. At anthesis, fresh pollen was collected in the morning by shaking the tassel in a plastic bag, while old pollen and anthers were removed from tassels by vigorous shaking the evening of the day before. For RNA isolation and qRT-PCR, four tissues (leaf, stem, root, and pollen) were collected from *P. deltoids*. Samples were frozen immediately in liquid nitrogen, and stored at −80°C for further analysis. Three biological replicates were performed.

### Protein extraction

The pollen samples (~0.3 g) were ground to a fine powder in a pestle and mortar in liquid nitrogen, and extracted with acetone containing 10% (w/v) TCA (for electrophoresis, Sigma-Aldrich) and 1% (w/v) DTT (biotechnology grade, Amersco). The samples were kept at −20°C for at least 2 h. The samples were centrifuged at 25,000 g for 20 min at 4°C, and the resulting pellets were washed by suspending in acetone containing 1% (w/v) DTT, incubated at −20°C for 2 h, and centrifuged as above. The pellets were suspended again in acetone, sonicated (15 s duration, 3 times with 5 min intervals) on ice at 200 W in 6 mm ultrasonic probe in JY92-II DN sonicator homogenizer (Ningbo Scientz Biotechnology Co, China), and centrifuged at 25,000 g as above. The pellets were vacuum dried and total soluble proteins were extracted by dissolving in isoelectric focusing (IEF) compatible buffer comprising 8 M urea, 20 mM DTT, 4% (w/v) CHAPS (ultrapure bioreagent, Sigma), and 2% (v/v) ampholytes (pH 4–7, GE Healthcare). Solution were vortexed extensively for 1 h at room temperature, centrifuged at 4°C for 20 min at 25,000 g, and the supernatants were collected. The resulting pellets were resolubilized and vortexed for 1 h, centrifuged at 25,000 g (20 min, 4°C), and the supernatants combined with those collected earlier. The resulting protein samples were centrifuged again for 20 min at 25,000 g (4°C). Total soluble protein in the supernatants was estimated with Bio-Rad protein assay (Bio-Rad, Hercules, CA, USA) and used immediately for further analysis or stored at −80°C for later use.

### Two-dimensional gel electrophoresis (2-DE)

2-DE was carried out as previously described (Sheoran et al., 2006). IEF was performed using the Ettan III system (GE Healthcare) and 18-cm Immobiline Dry Strips of 4–7 linear pH gradients (GE Healthcare, OK, USA). The strips were rehydrated overnight in a solution containing 8 M urea, 2% (w/v) CHAPS, 20 mM DTT, 0.002% (w/v) bromophenol blue, 2% (w/v) IPG buffer (pH 4–7), and 600 μg of the protein sample. IEF was carried out by applying a voltage of 250 V for 1 h, followed by an increase to 3500 V over 2 h, and holding at 8000 V until a total of 60 kVh was obtained.

Following IEF, the strips were equilibrated for 15 min in a buffer containing 0.1 M Tris-HCl (pH 8.8), 2% (w/v) SDS (proteomics grade, Amresco), 6 M urea, 30% (v/v) glycerol (biotechnology grade, Amersco) and 0.1 M DTT, and for another 15 min in the same buffer containing 0.25 M iodoacetamide without DTT. The equilibrated strips were applied to vertical SDS–polyacrylamide gels (12.5% resolving 5% stacking) and sealed with 0.5% agarose in SDS buffer (see below for the composition) containing bromophenol blue. Electrophoresis was performed in two gels for 30 min at 10 mA gel^−1^, and then at 30 mA gel^−1^ until the dye front reached the bottom of the gels, in an SDS electrophoresis buffer containing 25 mM TRIS base, 192 mM glycine, and 0.1% (w/v) SDS, pH 8.3 in a PROTEAN II XL multi-cell (Bio-Rad, USA).

### Gel staining and image analysis

Gels were fixed overnight in 50% (v/v) ethanol with 10% (v/v) orthophosphoric acid, washed with water (1 h), and stained with Colloidal Coomassie Blue G-250 (CCB) as described earlier (Sheoran et al., 2006). Images of the stained gels were captured with a scanner (UMAX Powerlook 2100 XL; UMAX, Taiwan, China) and analyzed with ImageMaster 2D Platinum Software (Version 6.0; Amersham Biosciences, Uppsala, Sweden). Two replicate gels were run for each of three different pooled pollen samples collected from different batches of plants.

### MALDI-TOF/TOF MS/MS

Selected spots were excised manually from the 2-DE gels and automatically de-stained, dehydrated, reduced with DTT, alkylated with iodoacetamide, and digested with gold grade trypsin (Mass grade, Promega) using a MassPREP protein digest station (Micromass, Manchester, UK) according to the recommended procedure. Samples were then analyzed by a MALDI-TOF/TOF tandem mass spectrometer ABI 4800 proteomics analyzer (Applied Biosystems, Framingham, MN). For acquisition of mass spectra, 0.4 μl samples were spotted onto a MALDI plate, followed by 0.4 μl matrix solution [0.5 M CHCA in 50% (v/v) ACN (HPLC grade, Fisher) and 0.05% (v/v) TFA (HPLC grade, Merck)]. Mass data were acquired with 4000 Series Explorer Software v3.5 in batch-processing mode of MS/MS. All MS survey scans were acquired over the mass range *m/z* 800–4000 in the reflection positive-ion mode. The MS peaks were detected on minimum S/N ratio ≥10 and cluster area S/N threshold ≥40 without smoothing and raw spectrum filtering. Peptide precursor ions corresponding to contaminants including keratin and the trypsin autolytic products were excluded in a mass tolerance of 0.5 Da.

### Database search, annotation, and allergen prediction

For protein identification, the acquired MS/MS data were uploaded on the Protein Pilot software (Applied Biosystems, Framingham, MN) and compared against *P. trichocarpa* genome database (http://phytozome.jgi.doe.gov/pz/portal.html#!info?alias=Org_Ptrichocarpa), NCBI non-redundant protein sequence database (NCBI-nr) and Swiss-Prot database. Searches were performed using the following parameters: trypsin as the proteolytic enzyme, allowing for one missed cleavage; carbamidomethylation of cysteine as a fixed modification; oxidation of methionine as a variable modification. Proteins identified with a Mowse score greater than 60 (significant at 95% confidence interval) are reported.

To annotate the identified proteins with Gene Ontology (GO) terms, the sequences were imported into Blast2GO (Conesa et al., 2005), a software package that retrieves GO terms, allowing gene functions to be determined and compared. These GO terms are assigned to query sequences, producing a broad overview of groups of genes catalogs into three ontology vocabularies, biological processes (BP), molecular functions (MF), and cellular components (CC). The output GO terms were then slimmed in REVIGO and treemaps were produced (Supek et al., 2011).

Allergen prediction were realized by using the SDAP-Structural Database of Allergenic Proteins (http://fermi.utmb.edu/SDAP/index.html) under the two conditions of (1) sequence similarity >35% between presently obtained proteins and reported allergen proteins and (2) the presence of at least eight consecutive amino acids in the analyzed protein sequences compared to known allergen proteins (Ivanciuc et al., 2003). Furthermore, predictions for antigenicity were realized using the online software (http://imed.med.ucm.es/Tools/antigenic.pl) based on the algorithm of Kolaskar and Tongaonkar (1990). By these criteria, some of the presently analyzed poplar mature pollen proteins were declared as likely corresponds to allergen related proteins.

### Publicly available microarray data analyses

Microarray data for various tissues were available at NCBI Gene Expression Omnibus (GEO) database (http://www.ncibi.nlm.nih.gow/geo/), notably under the series accession number GSE21481 (for *P. trichocarpa*). Probe sets corresponding to selected genes were identified using the online Probe Match tool POParray (http://aspendb.uga.edu/poparray). For genes with more than one probe sets, the median of expression values was considered. The expression data were normalized by the Gene Chip Robust Multiarray Analysis (GCRMA) algorithm followed by log transformation and average calculation. Normalized values were extracted for further analyses.

### Sequence alignments and phylogenetic analyses

Multiple alignment of profilin protein sequences from poplar, *Arabidopsis*, and rice were performed using the Clustal X2.1 program (Larkin et al., 2007). Phylogenetic trees were constructed using the neighbor-joining method in the MEGA package V5.2 (Tamura et al., 2011) with bootstrap values from 1000 replicates indicated at each node. Secondary structures of proteins were predicted using the Protein Structure Prediction Server (PRIPRED, http://bioinf.cs.ucl.ac.uk/psipred/).

### RNA isolation and real-time qRT-PCR

Total RNA was extracted using the RNeasy Plant Mini Kit (Qiagen) with on-column treatment using RNase-free DNase I (Qiagen) to remove any contamination of genomic DNA. First-strand cDNA synthesis was carried out with approximately 1 μg RNA using the SuperScript III reverse transcription kit (Invitrogen) and random primers according to the manufacturer's procedure. Primers with melting temperatures of 58–60°C and amplicon lengths of 100–250 bp were designed using Primer3 software (http://frodo.wi.mit.edu/primer3/input.htm). All primer sequences used are listed in Table S1. qRT-PCR was conducted on LightCycler 480 Detection System (Roche, Penzberg, Germany) using SYBR Premix Taq Kit (TaKaRa, Dalian, China) according to the manufacturer's instructions. The *PtActin* gene was used as internal control.

## Results and discussion

### Proteomic maps of P. deltoides mature pollen

**Figure 1:**
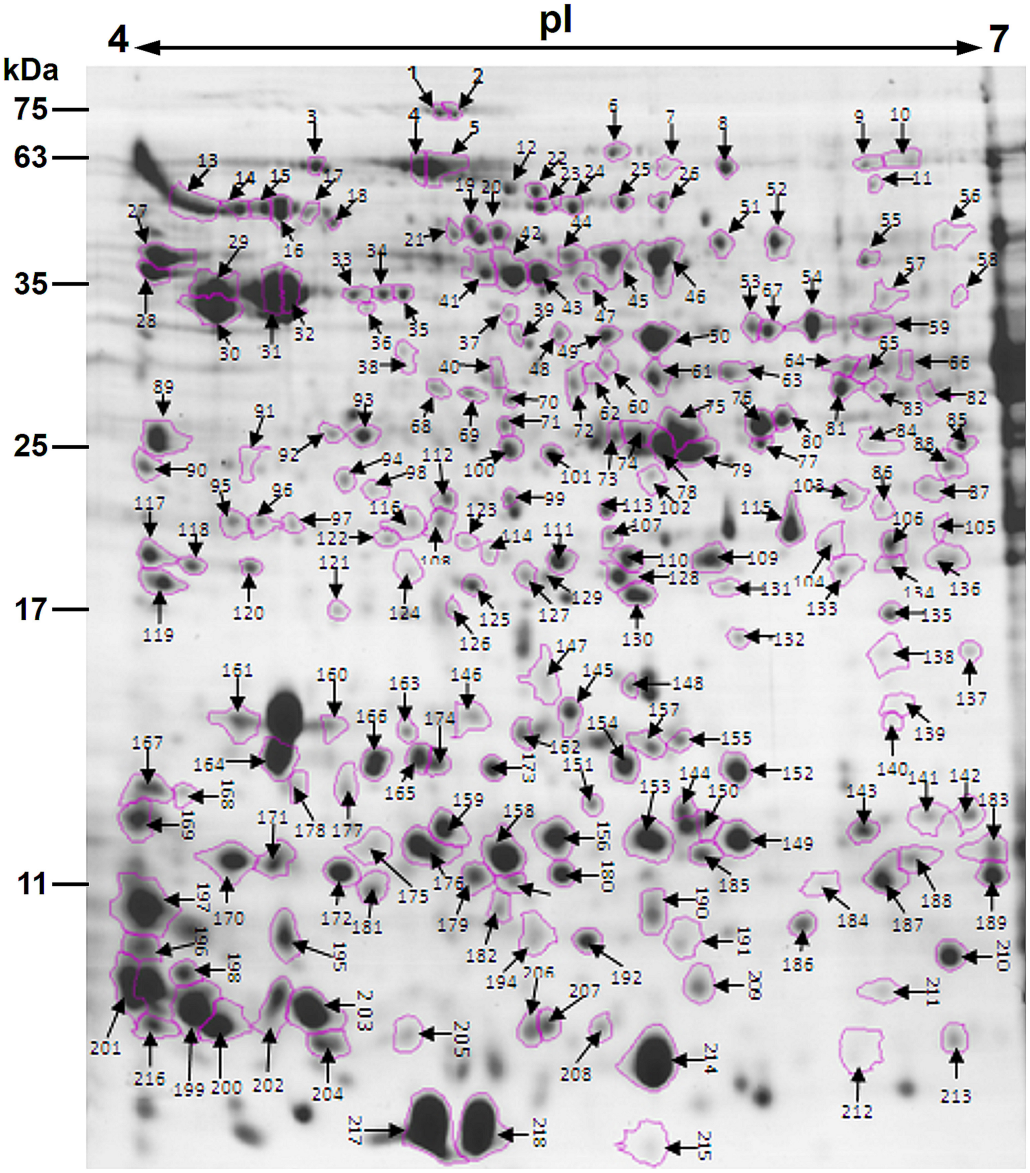
**Colloidal Coomassie stained 2-DE gel (pH 4–7) of *P. deltoides* CL. “2KEN8” mature pollen**. Spot numbers indicated on the gel were subjected to MALDI-TOF/TOF MS/MS analysis. Standard molecular weight markers are shown on the left.

After the 2-DE gels were aligned and matched, a total of 403 reproducible protein spots were detected in *P. deltoides* CL. “2KEN8” mature pollen. These proteins cover the pI range from 4 to 7 and the MW range from 5 to 120 kDa (Figure 1). All detected protein spots were processed by automated in-gel tryptic digestion and MALDI-TOF/TOF MS/MS analysis. After searching various publicly available protein databases, out of the 403 detected protein spots, 218 spots allowed the identification of 178 different proteins. Table 1 lists each of the identified proteins by its PACid number and corresponding *P. trichocarpa* gene locus, as obtained from Phytozome (http://phytozome.jgi.doe.gov/pz/portal.html#!info?alias=Org_Ptrichocarpa). The calculated MW of the identified proteins ranged from 6.9 to 106.0 kDa, and the calculated pI ranged from 4.26 to 8.95, which is close to the experimental data as judged from the location of the spots on the 2-DE gels (Table 1 and Figure 1).

**Table 1:** *****Populus deltoides*** CL. “2KEN8” mature pollen proteins identified by MALDI-TOF/TOF MS/MS**.

**Spot no**.	**PACid**	**Transcript Name**	**Protein description**	**Mw/pI**	**Cov (%)**	**Score**	***E*-value**	Proteins in pollen of of other species^*^
**CELL FATE (3, 1.35%)**								
27	27027720	Potri.005G015100.4	Calreticulin 1a	44/4.6	39	292	4.6E-25	b, d
28	26994433	Potri.013G009500.1	Calreticulin 1b	41.7/4.7	50	152	1.8E-12	b, d
86	27041363	Potri.001G112000.1	Nucleotide-rhamnose synthase/epimerase-reductase	34.1/5.9	53	231	5.8E-19	b, d
**CYTOSKELETON (14, 6.31%)**								
7	26992950	Potri.004G073900.1	Pectin lyase-like superfamily protein	52.2/6.1	30	129	9.2E-09	a, b
8	26992950	Potri.004G073900.1	Pectin lyase-like superfamily protein	52.2/6.1	33	125	2.3E-08	a, b
61	26989474	Potri.004G117800.1	Reversibly glycosylated polypeptide 2	41.8/5.7	69	280	7.3E-24	a, b, c, d
62	26989474	Potri.004G117800.1	Reversibly glycosylated polypeptide 2	41.8/5.7	45	60	6.5E-03	a, b, c, d
63	26985047	Potri.017G099100.1	Reversibly glycosylated polypeptide 2	41.8/5.8	52	154	2.9E-11	a, b, c, d
64	26985047	Potri.017G099100.1	Reversibly glycosylated polypeptide 2	41.8/5.8	60	182	4.6E-14	a, b, c, d
66	27024891	Potri.019G067200.1	Pectin lyase-like superfamily protein	42.4/8.1	32	100	7.3E-06	
80	27049793	Potri.012G114900.1	Pectin lyase-like superfamily protein	39.8/5.6	57	663	3.7E-62	b
177	27045539	Potri.001G127500.1	Plant invertase/pectin methylesterase inhibitor superfamily protein	19.6/5	30	71	5.5E-03	a, c, d
178	27045539	Potri.001G127500.1	Plant invertase/pectin methylesterase inhibitor superfamily protein	19.6/5	40	70	7.6E-03	a, c, d
200	27000020	Potri.003G047700.1	Profilin 3	14.2/4.7	58	411	5.8E-37	a, c
201	27004517	Potri.006G235200.2	Profilin 4	22.2/4.8	45	85	2.3E-04	a, b, c, d
203	27047644	Potri.001G190800.2	Profilin 5	14.2/4.8	51	132	4.6E-09	a, c, d
216	27011208	Potri.018G057600.7	Profilin 4	9.9/4.8	83	91	6.2E-05	a, b, c, d
**DEFENSE/STRESS RESPONSE (18, 8.11%)**								
9	26998620	Potri.003G113400.1	Stress-inducible protein, putative	65.8/6.2	39	106	1.8E-05	
10	26998620	Potri.003G113400.1	Stress-inducible protein, putative	65.8/6.2	46	355	2.3E-31	
118	27050381	Potri.012G061600.1	Glycine-rich RNA-binding protein 3	26.5/4.8	34	216	1.8E-17	c
119	27018937	Potri.015G057400.1	Glycine-rich RNA-binding protein 3	24.9/5	16	152	4.6E-11	c
131	26980674	Potri.010G211600.2	Dehydroascorbate reductase 2	23.7/5.8	75	299	9.2E-26	b, d
135	26995979	Potri.013G092600.1	Manganese superoxide dismutase 1	25.3/6.8	67	297	1.5E-25	a
141	27047492	Potri.001G105200.2	Glutathione peroxidase 6	17.5/7.6	51	63	3.8E-03	a, b, d
155	26986220	Potri.009G132000.1	Cold shock domain protein 1	17.4/5.6	53	157	1.5E-11	
157	26986372	Potri.009G007200.4	Lactoylglutathione lyase family protein/glyoxalase I family protein	20.9/5.6	67	167	1.5E-12	a, b, d
158	27009533	Potri.018G083500.1	Thioredoxin-dependent peroxidase 1	17.5/5.6	74	467	1.5E-42	a, d
162	26990347	Potri.004G172600.1	Cold shock domain protein 1	19.4/6.3	57	242	4.6E-20	c
168	27020577	Potri.002G251200.1	Lactoylglutathione lyase/glyoxalase I family protein	17.5/4.6	65	272	4.6E-23	a, b, d
182	26990766	Potri.004G155300.1	Cold, circadian rhythm, and RNA binding 1	16.6/5.5	75	68	1.2E-03	c
185	26995574	Potri.013G031100.1	Copper/zinc superoxide dismutase 1	21.1/7.3	35	154	2.9E-11	a, c
186	27030159	Potri.005G044400.3	Copper/zinc superoxide dismutase 1	14.1/5.9	60	123	3.7E-06	a, c
192	27009651	Potri.018G133400.1	Glutaredoxin family protein	14.9/5.8	58	62	4.9E-03	b, c
193	26999669	Potri.003G060600.3	Glutaredoxin 4	19.3/7.8	73	286	1.8E-24	b, c
195	27051476	Potri.T162000.3	Glycine-rich RNA-binding protein 3	12.5/5	85	189	9.2E-15	c
**DEVELOPMENT (13, 5.86%)**								
29	27015922	Potri.007G024000.1	Late embryogenesis abundant (LEA) protein	45/4.6	40	101	5.8E-06	
30	27015922	Potri.007G024000.1	Late embryogenesis abundant (LEA) protein	45/4.6	44	162	4.6E-02	
31	27031280	Potri.005G122400.1	Late embryogenesis abundant (LEA) protein	44.4/4.8	57	721	5.8E-68	
32	27031280	Potri.005G122400.1	Late embryogenesis abundant (LEA) protein	44.4/4.8	47	124	2.9E-08	
98	27028208	Potri.005G048000.1	Seed maturation protein	27.7/5.3	40	98	1.3E-05	
99	27028208	Potri.005G048000.1	Seed maturation protein	27.7/5.3	49	131	5.8E-09	
112	27028209	Potri.005G048000.2	Seed maturation protein	21.8/4.9	52	80	7.0E-04	
113	27028209	Potri.005G048000.2	Seed maturation protein	21.8/4.9	46	100	7.5E-06	
198	26983086	Potri.010G062800.1	Late embryogenesis abundant protein (LEA) family protein	9.7/5.5	37	179	9.2E-14	b
199	27047611	Potri.001G172900.1	Late embryogenesis abundant protein-related/LEA protein-related	11.1/4.7	43	109	9.2E-07	
202	27021334	Potri.002G006000.1	Late embryogenesis abundant protein, group 6	9.9/5.1	38	74	3.2E-03	
217	26983916	Potri.017G108400.1	Late embryogenesis abundant protein (LEA) family protein	6.9/6.1	80	124	2.9E-08	
218	26989389	Potri.004G107800.1	Late embryogenesis abundant protein (LEA) family protein	7.1/6.2	64	73	3.7E-03	
**ENERGY (41, 18.47%)**								
6	26990681	Potri.004G082800.1	NADH-ubiquinone dehydrogenase, mitochondrial, putative	80.9/5.9	39	86	2.1E-04	a, d
11	27017058	Potri.007G026400.4	Succinate dehydrogenase 1-1	70.6/6.4	58	371	5.8E-33	b
23	27011563	Potri.016G142900.2	Phosphoglycerate mutase, 2,3-bisphosphoglycerate-independent	61.4/5.4	38	128	1.2E-08	c, d
24	27011563	Potri.016G142900.2	Phosphoglycerate mutase, 2,3-bisphosphoglycerate-independent	61.4/5.4	47	136	1.8E-09	c, d
25	27005580	Potri.006G113300.1	Phosphoglycerate mutase, 2,3-bisphosphoglycerate-independent	61.1/5.4	47	220	7.3E-18	a, b, c, d
26	27005580	Potri.006G113300.1	Phosphoglycerate mutase, 2,3-bisphosphoglycerate-independent	61.1/5.4	45	132	4.6E-09	a, b, c, d
41	27039927	Potri.008G126600.1	ATP synthase alpha/beta family protein	60/5.9	55	154	2.9E-11	a, b, c, d
42	27039927	Potri.008G126600.1	ATP synthase alpha/beta family protein	60/5.9	66	241	5.8E-20	a, b, c, d
43	26982343	Potri.010G116600.3	ATP synthase alpha/beta family protein	60.3/6.1	67	618	1.2E-57	a, b, c, d
44	27004351	Potri.006G116800.1	Enolase	47.8/5.6	55	90	7.6E-05	a, b, c, d
45	27004351	Potri.006G116800.1	Enolase	47.8/5.6	60	175	2.3E-13	a, b, c, d
46	27004351	Potri.006G116800.1	Enolase	47.8/5.6	60	458	1.2E-41	a, b, c, d
51	27019637	Potri.015G131100.3	Enolase	47.7/5.7	68	290	7.3E-25	a, b, c, d
52	27019637	Potri.015G131100.3	Enolase	47.7/5.7	71	184	2.9E-14	a, b, c, d
55	26984288	Potri.017G144700.1	UDP-glucose pyrophosphorylase 2	51.7/5.8	47	118	1.2E-07	a, b, c, d
72	26991183	Potri.004G074400.3	UDP-GLUCOSE PYROPHOSPHORYLASE 1	43.7/6.7	34	139	9.2E-10	a, c, d
75	27025643	Potri.019G063600.1	pfkB-like carbohydrate kinase family protein	35.3/5.8	56	115	2.3E-07	a, b, d
76	27025644	Potri.019G063600.2	pfkB-like carbohydrate kinase family protein	35.2/5.8	70	627	1.5E-58	a, b, d
77	27025644	Potri.019G063600.2	pfkB-like carbohydrate kinase family protein	35.2/5.8	53	166	1.8E-12	a, b, d
81	27038014	Potri.008G166800.2	Lactate/malate dehydrogenase family protein	36.1/6.1	69	140	7.3E-10	a, b, d
82	27038014	Potri.008G166800.2	Lactate/malate dehydrogenase family protein	36.1/6.1	49	67	1.3E-03	a, b, d
84	26982650	Potri.010G117900.1	Aldolase superfamily protein	42.8/8.4	27	61	6.4E-03	a, c, d
89	27029430	Potri.005G162100.1	Cytochrome C oxidase 6B	21.2/4.4	48	160	7.3E-12	c
92	26984355	Potri.017G029000.1	pfkB-like carbohydrate kinase family protein	35.5/4.9	60	167	1.5E-12	a, b, d
93	26984355	Potri.017G029000.1	pfkB-like carbohydrate kinase family protein	35.5/5	66	156	1.8E-11	a, b, d
100	27047658	Potri.001G061400.4	Transketolase family protein	38.8/5.9	51	95	2.1E-05	b, c, d
101	27047658	Potri.001G061400.4	Transketolase family protein	38.8/5.9	51	396	1.8E-35	b, c, d
104	27015019	Potri.007G079500.1	Copper ion binding; cobalt ion binding; zinc ion binding	27.8/8.5	58	467	1.5E-42	b, d
110	27029145	Potri.005G085500.2	Copper ion binding; cobalt ion binding; zinc ion binding	28/7.7	61	262	4.6E-22	b, d
111	27029145	Potri.005G085500.2	Copper ion binding; cobalt ion binding; zinc ion binding	28/7.7	53	217	1.5E-17	b, d
122	27040209	Potri.001G147300.1	Haloacid dehalogenase-like hydrolase (HAD) superfamily protein	28.1/5	48	65	2.5E-03	b
123	27040209	Potri.001G147300.1	Haloacid dehalogenase-like hydrolase (HAD) superfamily protein	28.1/5	44	104	2.9E-06	b
124	26997068	Potri.003G086900.1	Haloacid dehalogenase-like hydrolase (HAD) superfamily protein	27.5/4.9	31	86	2.0E-04	
133	27039000	Potri.008G056300.4	Triosephosphate isomerase	23.6/6	54	483	3.7E-44	b, c, d
133	27038998	Potri.008G056300.2	Triosephosphate isomerase	27.5/6	65	327	1.5E-28	b, c, d
145	26999999	Potri.003G086100.1	ATPase, F1 complex, delta/epsilon subunit	22.2/6.5	24	178	1.2E-13	a, d
159	26980006	Potri.010G217800.1	ATP synthase D chain, mitochondrial	19.6/5.2	81	272	4.6E-23	a, b, d
170	27046945	Potri.001G173800.1	Rubredoxin-like superfamily protein	18.9/5.3	75	173	3.7E-13	c
175	27036443	Potri.008G043800.1	ATP synthase D chain, mitochondrial	19.9/5.1	68	130	7.3E-09	a, b, d
176	27036443	Potri.008G043800.1	ATP synthase D chain, mitochondrial	19.9/5.1	79	551	5.8E-51	a, b, d
207	26995508	Potri.013G108300.2	Vacuolar ATP synthase subunit G2	12.2/5.5	58	93	3.3E-05	a
**METABOLISM (26, 11.71%)**								
19	26997206	Potri.003G072600.1	Alanine aminotransferase 2	59/5.8	42	181	5.8E-14	
38	26978978	Potri.010G224300.3	Adenosine kinase 2	35.8/5.7	61	203	3.7E-16	b, d
48	27038006	Potri.008G099300.1	*S*-adenosylmethionine synthetase family protein	43.6/5.5	43	142	4.6E-10	a, b, c, d
49	27038006	Potri.008G099300.1	*S*-adenosylmethionine synthetase family protein	43.6/5.5	39	143	3.7E-10	a, b, c, d
50	27038006	Potri.008G099300.1	*S*-adenosylmethionine synthetase family protein	43.6/5.5	79	483	3.7E-44	a, b, c, d
53	27003913	Potri.006G123200.1	Methionine adenosyltransferase 3	43/5.8	57	130	7.3E-09	a, b, c, d
54	27003913	Potri.006G123200.1	Methionine adenosyltransferase 3	43/5.8	65	617	1.5E-57	a, b, c, d
58	27048527	Potri.001G420300.1	Tryptophan synthase beta-subunit 2	51.8/6.7	27	84	2.8E-04	
60	27016371	Potri.007G069600.2	Glutamine synthase clone R1	32.9/5.9	25	75	2.5E-03	c
67	27021791	Potri.002G189000.1	*S*-adenosylmethionine synthetase 2	43.6/5.6	47	72	4.2E-03	a, b, c, d
83	27050857	Potri.012G045900.1	Galactose mutarotase-like superfamily protein	36.5/7.1	52	82	5.2E-04	b, d
87	27012457	Potri.016G116400.2	Aldolase-type TIM barrel family protein	23.7/6.5	34	80	7.1E-04	b, d
94	26987714	Potri.009G121300.2	Papain family cysteine protease	41/5.8	17	60	6.5E-03	
102	26993311	Potri.T174200.1	Chorismate mutase 2	28.9/5.5	30	62	4.2E-03	
105	27023145	Potri.002G034100.1	Gamma carbonic anhydrase 1	31.3/6.7	53	314	2.9E-27	b, d
106	26989473	Potri.004G135300.1	Catalytic LigB subunit of aromatic ring-opening dioxygenase family	29.7/5.9	69	171	5.8E-13	
107	27033421	Potri.014G107100.1	Pyrophosphorylase 1	25/5.7	50	316	1.8E-27	a, b, c, d
115	27020835	Potri.002G181300.1	Pyrophosphorylase 1	24.9/5.9	46	400	7.3E-36	d
121	27045723	Potri.001G025700.1	Beta-galactosidase 7	93.2/5	14	90	6.7E-05	
136	27005436	Potri.006G082500.3	Pyrophosphorylase 4	24.9/5.9	48	322	4.6E-28	a, b, c, d
154	27020768	Potri.002G134600.6	P-loop containing nucleoside triphosphate hydrolases superfamily protein	22.2/5.5	41	63	4.0E-03	a, b, d
173	27035250	Potri.014G043300.1	P-loop containing nucleoside triphosphate hydrolases superfamily protein	23/5.6	66	191	5.8E-15	a, b, d
174	27035250	Potri.014G043300.1	P-loop containing nucleoside triphosphate hydrolases superfamily protein	23/5.6	91	434	2.9E-39	a, b, d
190	26998708	Potri.003G107100.1	Lipase/lipooxygenase, PLAT/LH2 family protein	20.4/6.1	48	139	9.2E-10	
210	27033722	Potri.014G049900.1	Nucleoside diphosphate kinase family protein	16.3/6.1	58	219	9.2E-18	d
214	26996661	Potri.003G103700.3	Acyl-CoA-binding protein 6	10.1/5.4	67	117	1.5E-07	
**PROTEIN FATE (39, 17.57%)**								
4	27042356	Potri.001G087500.1	Heat shock protein 70 (Hsp 70) family protein	73.8/5.1	46	550	7.3E-51	a, c, d
5	27042356	Potri.001G087500.1	Heat shock protein 70 (Hsp 70) family protein	73.8/5.1	45	327	1.5E-28	a, c, d
12	27041993	Potri.001G285500.1	Mitochondrial HSO70 2	73.4/5.6	37	203	3.7E-16	b, d
14	27031716	Potri.005G179000.1	PDI-like 1-1	55.2/4.7	35	115	2.3E-07	b, d
15	27031716	Potri.005G179000.1	PDI-like 1-1	55.2/4.7	41	120	7.3E-08	b, d
16	27024556	Potri.002G082100.1	PDI-like 1-2	56.4/4.8	62	478	1.2E-43	
17	27024556	Potri.002G082100.1	PDI-like 1-2	56.4/4.8	50	291	5.8E-25	
18	26992579	Potri.004G213400.1	Chaperonin-60alpha	62.3/5.2	39	96	2.0E-05	c
20	26997267	Potri.003G173900.1	Heat shock protein 60	61.5/5.8	53	175	2.3E-13	b, d
21	26997267	Potri.003G173900.1	Heat shock protein 60	61.5/5.8	48	463	3.7E-42	b, d
22	26987596	Potri.009G079700.1	Mitochondrial HSO70 2	73.4/5.6	37	105	2.3E-06	b, d
40	27049839	Potri.012G014100.1	HSP20-like chaperones superfamily protein	33.9/5.9	45	86	1.6E-04	
68	27019704	Potri.015G013900.1	HSP20-like chaperones superfamily protein	34.5/5.3	62	222	4.6E-18	
69	27019704	Potri.015G013900.1	HSP20-like chaperones superfamily protein	34.5/5.3	53	161	5.8E-12	
70	27019704	Potri.015G013900.1	HSP20-like chaperones superfamily protein	34.5/5.3	34	112	4.6E-07	
79	27022705	Potri.002G198300.2	Thioredoxin family protein	39.9/5.6	35	260	7.3E-22	a, b, d
108	27002177	Potri.011G089000.2	Co-chaperone GrpE family protein	34.6/6	34	79	1.0E-04	
116	27002178	Potri.011G089000.3	Co-chaperone GrpE family protein	33.6/6	32	106	1.8E-06	
125	27008889	Potri.018G063200.2	Chaperonin 20	26.9/6.6	68	227	1.5E-18	b
130	27006870	Potri.006G138600.2	Chaperonin 20	26.9/8.8	54	190	7.3E-15	b
140	26979786	Potri.010G053400.2	Heat shock protein 21	27.2/8.9	53	103	3.7E-06	
142	26978464	Potri.010G195700.1	HSP20-like chaperones superfamily protein	18.6/6.2	58	219	9.2E-18	
143	27041037	Potri.001G254700.1	HSP20-like chaperones superfamily protein	16.5/5.7	39	134	2.9E-09	
144	27041037	Potri.001G254700.1	HSP20-like chaperones superfamily protein	16.5/5.7	37	101	5.8E-06	
147	27009726	Potri.018G145900.1	N-terminal nucleophile aminohydrolases (Ntn hydrolases) superfamily protein	25.8/5.4	45	105	2.3E-06	d
148	27009726	Potri.018G145900.1	N-terminal nucleophile aminohydrolases (Ntn hydrolases) superfamily protein	25.8/5.4	56	282	4.6E-24	d
150	26993072	Potri.004G187200.1	HSP20-like chaperones superfamily protein	13/4.5	46	202	4.6E-16	
151	26993072	Potri.004G187200.1	HSP20-like chaperones superfamily protein	13/5.5	36	121	5.8E-08	
152	26994024	Potri.013G089200.1	HSP20-like chaperones superfamily protein	21.9/5.9	64	192	4.6E-15	
156	26988666	Potri.009G147900.1	HSP20-like chaperones superfamily protein	18.6/5.2	58	129	9.2E-09	
165	26999653	Potri.003G109200.1	Mitochondrion-localized small heat shock protein 23.6	24/6.4	52	118	1.2E-07	
166	26999656	Potri.003G109200.4	Mitochondrion-localized small heat shock protein 23.6	18.7/5.3	52	189	9.2E-15	
171	26994600	Potri.013G102100.1	Thioredoxin superfamily protein	23.2/7.7	46	276	1.8E-23	
184	27006960	Potri.006G093500.1	HSP20-like chaperones superfamily protein	15.8/5.8	51	70	7.5E-03	
187	27005125	Potri.006G223900.1	17.6 kDa class II heat shock protein	17.6/6.2	43	242	4.6E-20	
188	27000253	Potri.003G071100.1	HSP20-like chaperones superfamily protein	17.7/6.4	51	280	7.3E-24	
189	27024141	Potri.002G248200.1	FK506- and rapamycin-binding protein 15 kD-2	16.2/7.7	59	85	2.4E-04	d
205	27034980	Potri.014G044300.1	GroES-like family protein	15.1/8.9	52	100	7.3E-06	
208	27029466	Potri.005G232700.2	Thioredoxin H-type 1	10.4/6.8	47	126	1.8E-08	b
**PROTEIN SYNTHESIS AND PROCESSING (32, 14.41%)**								
1	27012288	Potri.016G091600.1	ATPase, AAA-type, CDC48 protein	90.1/5.1	44	187	1.5E-14	a, b, c
2	27003641	Potri.006G125500.2	ATPase, AAA-type, CDC48 protein	84.7/5	39	142	4.6E-10	a, b, c
3	26999807	Potri.003G006300.1	Chloroplast heat shock protein 70-2	75.4/5.2	47	593	3.7E-55	
33	27031369	Potri.005G025100.1	Regulatory particle triple-A ATPase 5A	47.6/4.9	58	224	2.9E-18	d
34	26995609	Potri.013G016800.1	Regulatory particle triple-A ATPase 5A	47.7/5	56	189	9.2E-15	d
35	26995609	Potri.013G016800.1	Regulatory particle triple-A ATPase 5A	47.7/5	66	243	3.7E-20	d
36	27050895	Potri.012G144700.1	Ribosomal protein S5 domain 2-like superfamily protein	46.2/5.3	48	65	2.5E-03	
47	27011934	Potri.016G028000.1	Regulatory particle triple-A ATPase 3	46.6/5.4	60	97	1.3E-05	d
59	27023547	Potri.002G215900.1	GTP binding elongation factor Tu family protein	49.3/7.7	43	70	8.0E-03	a, b, d
90	27017809	Potri.015G003300.2	Nascent polypeptide-associated complex subunit alpha-like protein 2	24.5/4.3	19	73	3.6E-03	
95	26988643	Potri.009G018600.1	Glutathione S-transferase, C-terminal-like; Translation elongation factor EF1B/ribosomal protein S6	24.6/4.6	56	130	7.3E-09	a, b, c, d
96	26997595	Potri.003G081000.4	Ubiquitin C-terminal hydrolase 3	21.6/4.8	43	313	3.7E-27	
97	27005563	Potri.006G141700.2	Cysteine proteinases superfamily protein	40/6.3	28	108	1.2E-06	
109	27005638	Potri.006G008800.1	20S proteasome alpha subunit C1	27.6/6	74	404	2.9E-36	
117	27042451	Potri.001G034400.1	Nascent polypeptide-associated complex (NAC), alpha subunit family protein	22.3/4.3	31	100	7.3E-06	
120	27045481	Potri.001G162900.1	20S proteasome alpha subunit E2	26.2/4.8	71	261	5.8E-22	
127	27018307	Potri.015G122400.1	Proteasome subunit PAB1	31.7/9	54	147	1.5E-10	
128	27018307	Potri.015G122400.1	Proteasome subunit PAB1	31.7/9	63	192	4.6E-15	
134	26983507	Potri.017G071100.1	20S proteasome beta subunit PBB2	30.2/6.8	29	68	1.2E-03	
137	27027528	Potri.005G092600.1	Nuclear-encoded CLP protease P7	27/8.7	38	167	1.5E-12	
149	27003950	Potri.006G185000.1	Eukaryotic elongation factor 5A-1	17.7/5.6	61	104	2.9E-06	b, d
153	26981508	Potri.010G162800.1	Eukaryotic elongation factor 5A-1	17.6/5.5	60	258	1.2E-21	b
160	27005899	Potri.006G087900.2	Ribosomal protein S7e family protein	17.9/10.5	50	74	1.70E-08	
167	27000087	Potri.003G010200.1	60S acidic ribosomal protein family	12.5/4.3	29	180	7.3E-14	a, d
169	27039265	Potri.008G092000.1	Eukaryotic elongation factor 5A-1	17.6/5.6	62	205	2.3E-16	b, d
179	27022821	Potri.002G056200.2	Ribosomal protein L7Ae/L30e/S12e/Gadd45 family protein	15.7/5.5	57	343	3.7E-30	a
180	27022821	Potri.002G056200.2	Ribosomal protein L7Ae/L30e/S12e/Gadd45 family protein	15.7/5.5	65	311	5.8E-27	a
183	27005768	Potri.006G205700.7	MMS ZWEI homolog 3	16.8/6.2	91	111	5.8E-07	c
196	26992109	Potri.004G118800.1	Ribosomal protein S19e family protein	15.9/10.2	73	107	1.5E-06	a
197	26985759	Potri.009G146200.1	60S acidic ribosomal protein family	11.4/4.4	30	127	1.5E-08	a, d
215a	27024233	Potri.002G062500.1	Related to ubiquitin 1	17.3/5.8	65	240	7.3E-20	
215b	27029355	Potri.005G198700.2	Related to ubiquitin 1	15.4/5.4	85	268	1.2E-22	a
**SIGNAL TRANSDUCTION (4, 1.80%)**								
73	27041495	Potri.001G174600.1	Transducin/WD40 repeat-like superfamily protein	38.4/5.6	43	67	1.4E-03	d
85	27020338	Potri.002G095600.1	Annexin 1	36.1/6.2	59	206	1.8E-16	b, d
103	27018481	Potri.015G068900.2	Transducin family protein/WD-40 repeat family protein	33/5.7	70	278	1.2E-23	d
138	27018218	Potri.015G129000.1	P-loop containing nucleoside triphosphate hydrolases superfamily protein	27.3/8.3	37	99	9.0E-06	a, d
**TRANSPORT (3, 1.35%)**								
206	27050254	Potri.012G039100.1	Tim10/DDP family zinc finger protein	11.2/5.8	40	161	5.8E-12	a, d
212	26986284	Potri.009G039600.1	Tim10/DDP family zinc finger protein	9.9/5.6	66	109	9.2E-07	a, d
213	26996993	Potri.003G170800.2	Nuclear transport factor 2A	12.8/6.2	53	155	2.3E-11	
**UNCLASSIFIED PROTEIN (29, 13.06%)**								
13	26991752	Potri.004G127400.3	Similar to latex abundant protein 1	43.9/4.6	27	396	1.8E-35	
37	27027243	Potri.T066800.1	RNA-binding (RRM/RBD/RNP motifs) family protein	47.3/5.3	34	195	2.3E-15	
39	27000938	Potri.011G138400.1	RNA-binding protein 45A	49.9/7.2	40	84	2.8E-04	
56a	27029928	Potri.005G094400.3	Seryl-tRNA synthetase/serine–tRNA ligase	28.3/8.5	70	81	5.7E-04	
56b	27029926	Potri.005G094400.1	Seryl-tRNA synthetase/serine–tRNA ligase	52.1/5.9	49	93	3.6E-05	
57	27045673	Potri.001G153000.1	Hyaluronan/mRNA binding family	39.8/6.3	28	65	2.1E-03	
65	27033257	Potri.014G067700.1	Arginase	36.6/5.8	54	79	9.2E-04	
71	27042241	Potri.001G182100.1	Pyridoxine biosynthesis 1.2	33.5/5.2	47	74	3.3E-03	
74	27037622	Potri.008G102900.1	RmlC-like cupins superfamily protein	38.9/5.6	48	140	7.3E-10	a, b, d
78	27020856	Potri.002G034400.1	NmrA-like negative transcriptional regulator family protein	34/5.5	60	280	7.3E-24	
88	26988646	Potri.009G105000.1	Alpha/beta-hydrolases superfamily protein	33.3/6	47	399	9.2E-36	
91a	26988410	Potri.009G073400.1	RAN binding protein 1	24.7/4.7	32	160	7.3E-12	
91b	27044312	Potri.001G278900.1	Ppeckstrin homology (PH) domain superfamily protein	24.3/4.7	31	214	2.9E-17	
114	26986988	Potri.009G096400.1	Domain of unknown function (DUF303)	27.5/5.3	43	61	6.1E-03	
126	27041780	Potri.001G347900.1	Polyribonucleotide nucleotidyltransferase, putative	106/6.5	20	60	6.5E-03	
129	27046848	Potri.001G332700.1	Aldolase-type TIM barrel family protein	24.5/5.2	22	99	9.2E-06	
132	26995635	Potri.013G057900.2	Adenosine-5'-phosphosulfate (APS) kinase 3	23.4/6	54	146	1.6E-10	
139	27007450	Potri.006G272400.1	Serine/arginine-rich 22	21.2/11.3	42	79	9.6E-04	
146	26979162	Potri.010G003500.1	Cytidine/deoxycytidylate deaminase family protein	20.7/5.3	59	90	7.1E-05	
161	27041887	Potri.001G392400.1	Pollen Ole e 1 allergen and extensin family protein	18.1/4.8	36	79	8.8E-04	
163	27028964	Potri.005G253600.1	Potri.005G253600.1	24.9/5.5	52	120	7.3E-08	
164	27001602	Potri.011G111300.1	Pollen Ole e 1 allergen and extensin family protein	18.3/4.9	51	483	3.7E-44	
172	26997047	Potri.003G060100.2	Rubredoxin-like superfamily protein	24.2/5.4	50	251	5.8E-21	
181	27015291	Potri.007G018000.1	Thioredoxin H-type 1	13.4/5.1	36	132	4.6E-09	b
191	27045934	Potri.001G237500.1	Dessication-induced 1VOC superfamily protein	16/6.1	43	104	2.9E-06	a, b, d
194	27012437	Potri.016G104600.3	Adenine nucleotide alpha hydrolases-like superfamily protein	16.5/5	47	98	1.3E-05	
204	27015578	Potri.007G138400.1	Bifunctional inhibitor/lipid-transfer protein/seed storage 2S albumin superfamily protein	13.6/5.5	25	102	4.6E-06	
209	26989811	Potri.004G022700.1	Tetratricopeptide repeat (TPR)-like superfamily protein	17.1/6.6	16	70	7.1E-03	
211	26988496	Potri.009G022300.1	Cystatin B	11.2/5.6	77	127	1.5E-08	

* *Proteins were identified in pollen of other species. a, Proteins were identified in Arabidopsis mature pollen using 2-DE and Electrospray ionization tandem mass spectrometry (ESI-MS/MS) by Holmes-Davis et al. (2005); b, Proteins were identified in Arabidopsis mature pollen using 2-DE in combination with MALDI-TOF MS and LC-MS/MS by Noir et al. (2005); c, Proteins were identified in tomato pollen using 2-DE and MALDI-TOF MS by Sheoran et al. (2007); d, Proteins were identified in Arabidopsis mature pollen and pollen tubes using 2-DE and MALDI-TOF MS by Zou et al. (2009)*.

Proteomic analyses showed that correlations between identified proteins and spot locations on the gels 2D-might not be one-to-one. In particular, expression of a given gene can give rise to several polypeptides located in different gel spots (Noir et al., 2005; Zou et al., 2009). Consistent with this, the present study showed that 40 proteins were associated with different spots (Table 1). There might be several possibilities to account for different electrophoretic migrations of the same protein in a 2D-gel. One possibility corresponds to post-translational modifications of the proteins, such as phosphorylation, methylation, glycosylation, or acetylation (Jensen, 2004). In general, these modifications do not significantly affect the MW of a protein, but induce a pI shift on the protein spot on the gel (Holmes-Davis et al., 2005; Noir et al., 2005). Another possibility relies on alternative splicing of mRNAs during translation (Brett et al., 2002) or on partial synthesis of proteins during pollen maturation (Loraine et al., 2013).

A previous study identified 135 proteins from *Arabidopsis* mature pollen proteome (Holmes-Davis et al., 2005), of which 74 were presently detected in *P. deltoids* mature pollen (Table 1). Another study identified 121 proteins from *Arabidopsis* mature pollen proteome (Noir et al., 2005), of which 86 were presently detected in *P. deltoids* mature pollen (Table 1). Zou et al. (2009) by using 2-DE and MALDI-TOF MS identified 189 distinct proteins from *Arabidopsis* mature pollen and pollen tubes, and 98 of them were detected in the present study (Table 1). In tomato, 133 proteins were identified from tomato pollen (Sheoran et al., 2007), and 56 of them were also detected in *P. deltoids* mature pollen in our present study. Therefore, despite differences that may be attributed to the methods of protein extraction, choices of immobilized pH gradient (IPG) strips with different pH ranges, spot selection for MS analysis and the different plant species used, our present data support the finding that the *P. deltoids* mature pollen proteome exhibits common features with mature pollen proteomes from other plant species. However, it is noted that in our study, 89 new proteins were identified that were not reported before in the previous studies mentioned above (Table 1). Most of these newly identified proteins were classified into development and protein fate. Interestingly, a large number of sHsps (spots No. 40, 68, 69, 70, 142, 143, 144, 150, 151, 152, 156, 165, 166, 184, 187, and 188) were identified in *P. deltoides* mature pollen. It has been reported that sHsp expression is developmentally induced in tobacco pollen. Notably, it appears that different subsets of cytosolic sHsp genes are expressed in a stage-specific fashion suggesting that certain sHsp genes may play specific roles in early stages of pollen development, while others may play a role in later stages, e.g., desiccation tolerance (Volkov et al., 2005). Moreover, we found that spots No. 96, 109, 120, 127, 128, and 134 are proteasome-related proteins, in agreement with the finding that the ubiquitin/proteasome pathway is involved in pollen tube growth (Sheng et al., 2006). Most of the protein fate proteins seem to correspond to Hsps. This presumably reflects a remnant process of late pollen development allowing desiccation tolerance. The majority of identified proteins are preferentially implicated in the determination of protein fate (17.57%) rather than in protein synthesis (14.41%, Table 1). It has been demonstrated that *Arabidopsis* pollen is charged with stored mRNA and performed translation apparatus enabling rapid activation upon hydration and germination (Honys and Twell, 2003) as it was established for seeds (Rajjou et al., 2004; Galland et al., 2014). Our results support this concept.

### Functional classification of identified proteins

**Figure 2:**
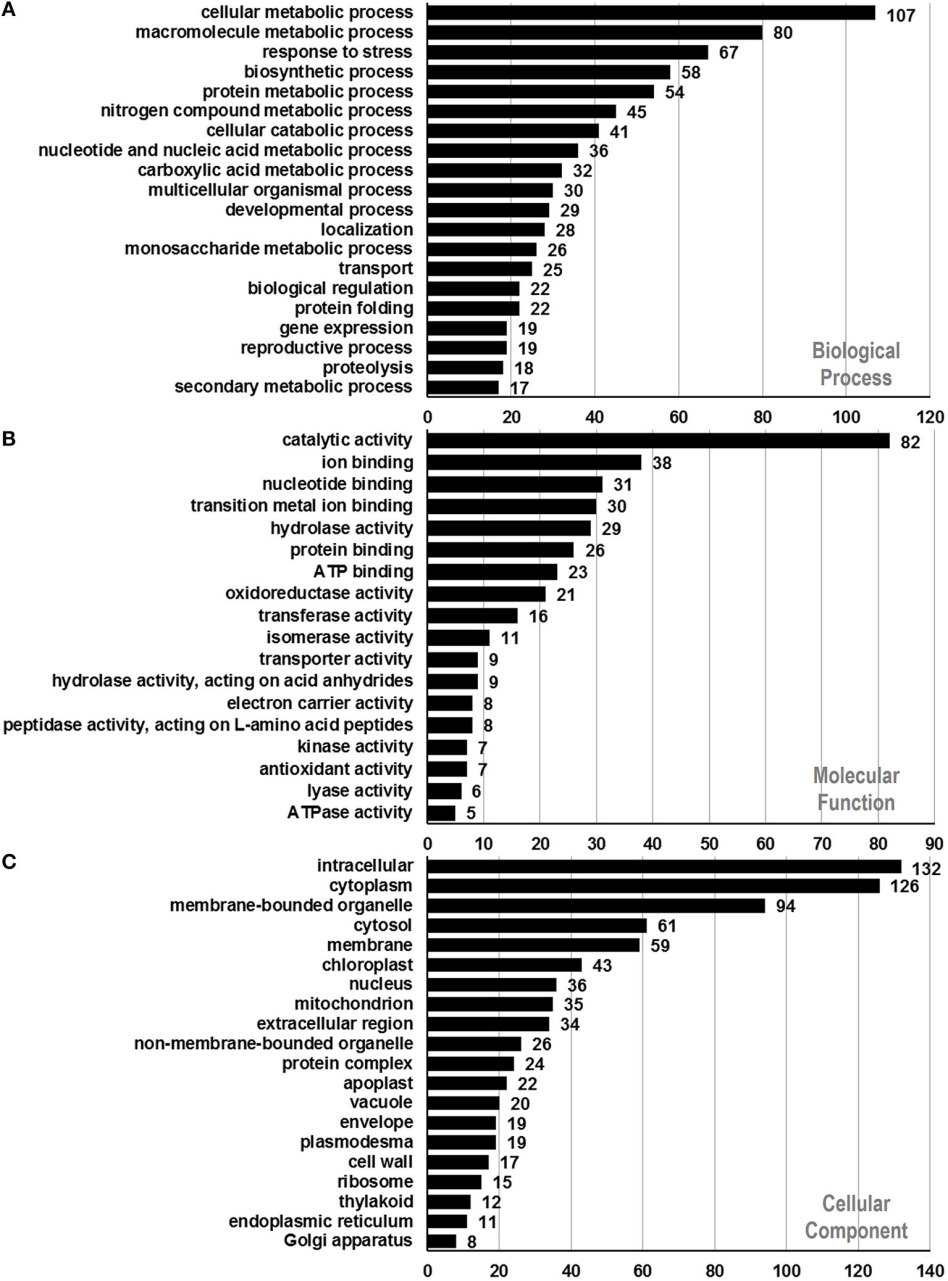
**Functional annotation of identified proteins based on GO categorization**. Results are summarized for three main GO categories. **(A)** Biological process, **(B)** molecular function, and **(C)** cellular component. The number corresponding to each column indicates the number of proteins in this GO term.

To assign functional information to the presently identified proteins, we first classified them into functional groups according to previous proteomic analyses (Holmes-Davis et al., 2005; Noir et al., 2005; Sheoran et al., 2007; Zou et al., 2009). As shown in Table 1, the three major groups of identified proteins in *P. deltoides* mature pollen were involved in energy regulation (18.47%), protein fate (17.57%), protein synthesis and processing (14.41%), and metabolism (11.71%) in good agreement with previous studies showing that the majority of proteins expressed in mature *Arabidopsis* pollen are involved in energy and general metabolism (Holmes-Davis et al., 2005; Noir et al., 2005; Sheoran et al., 2006). Furthermore, GO analysis was carried out, which provides a dynamic, controlled vocabulary, and hierarchical relationships for the representation of information on biological process (BP), molecular function (MF), and cellular component (CC), allowing a coherent annotation of genes and their products (Ashburner et al., 2000). For BP, cellular metabolic process (GO:0044237, 107 proteins) was the most represented GO term, followed by macromolecule metabolic process (GO:0043170, 80 proteins) and response to stress (GO:0006950, 67 proteins) (Figure 2A). Regarding MF, proteins with catalytic activity (GO:0003824, 82 proteins) and ion binding (GO:0043167, 38 proteins) were highly represented (Figure 2B). For CC, the most represented category was intracellular (GO:0005622, 132 proteins), cytoplasm (GO:0005737, 126 proteins), and membrane-bounded organelle (GO:0043227, 94 proteins) (Figure 2C).

**Figure 3:**
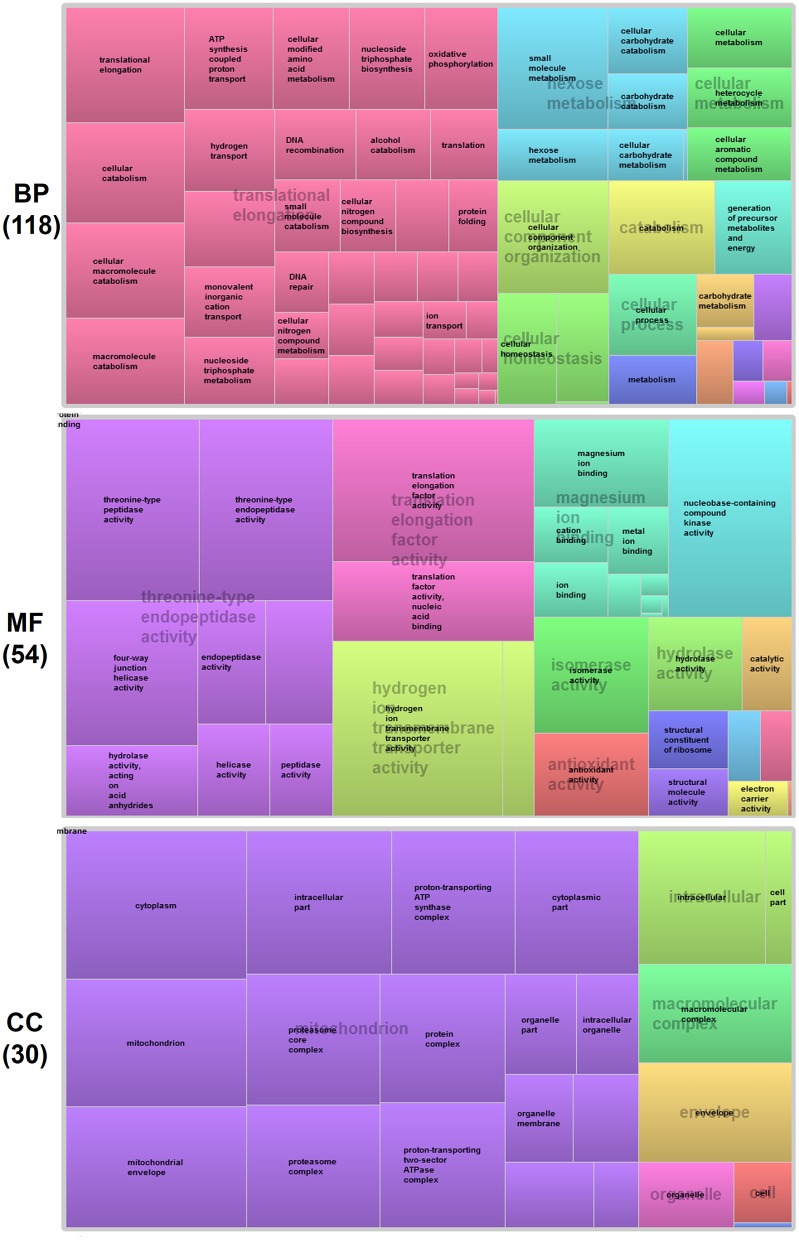
**GO treemaps for the identified proteins in *P. deltoides* pollen**. GO terms for proteins identified in *P. deltoides* pollen are shown. The box size correlates to the -log10 *P*-value of the GO-term. Boxes with the same color can be grouped together and correspond to the same upper-hierarchy GO-term which is found in the middle of each box.

To gain further insight into functional classification of proteins present in the *P. deltoides* mature pollen proteome, the GO terms along with their *P*-values were further summarized independently by the REVIGO reduction analysis tool that condenses the GO description by removing redundant terms (Supek et al., 2011). The results of these further reductions are visualized in Figure 3. For categories based on BP, the translational elongation and hexose metabolism processes were main GO terms in *P. deltoides* mature pollen. For categories based on CC, the identified proteins were mainly related with mitochondrial function.

### Prediction of allergens in *P. deltoides* mature pollen

Pollen grains are known to contain a number of proteins that can act as allergens (Mohapatra and Knox, 1996). Poplar trees release large amounts of pollen in spring that might cause the allergenic response. To date, many sequences and structures of allergenic proteins have been determined. Most of them can be grouped into a few families (Aalberse, 2000; Breiteneder and Ebner, 2000), suggesting that they share common characteristics that contribute to their ability to bind IgE and trigger an allergic reaction (Ipsen and Løwenstein, 1997; Sicherer, 2001).

**Figure 4:**
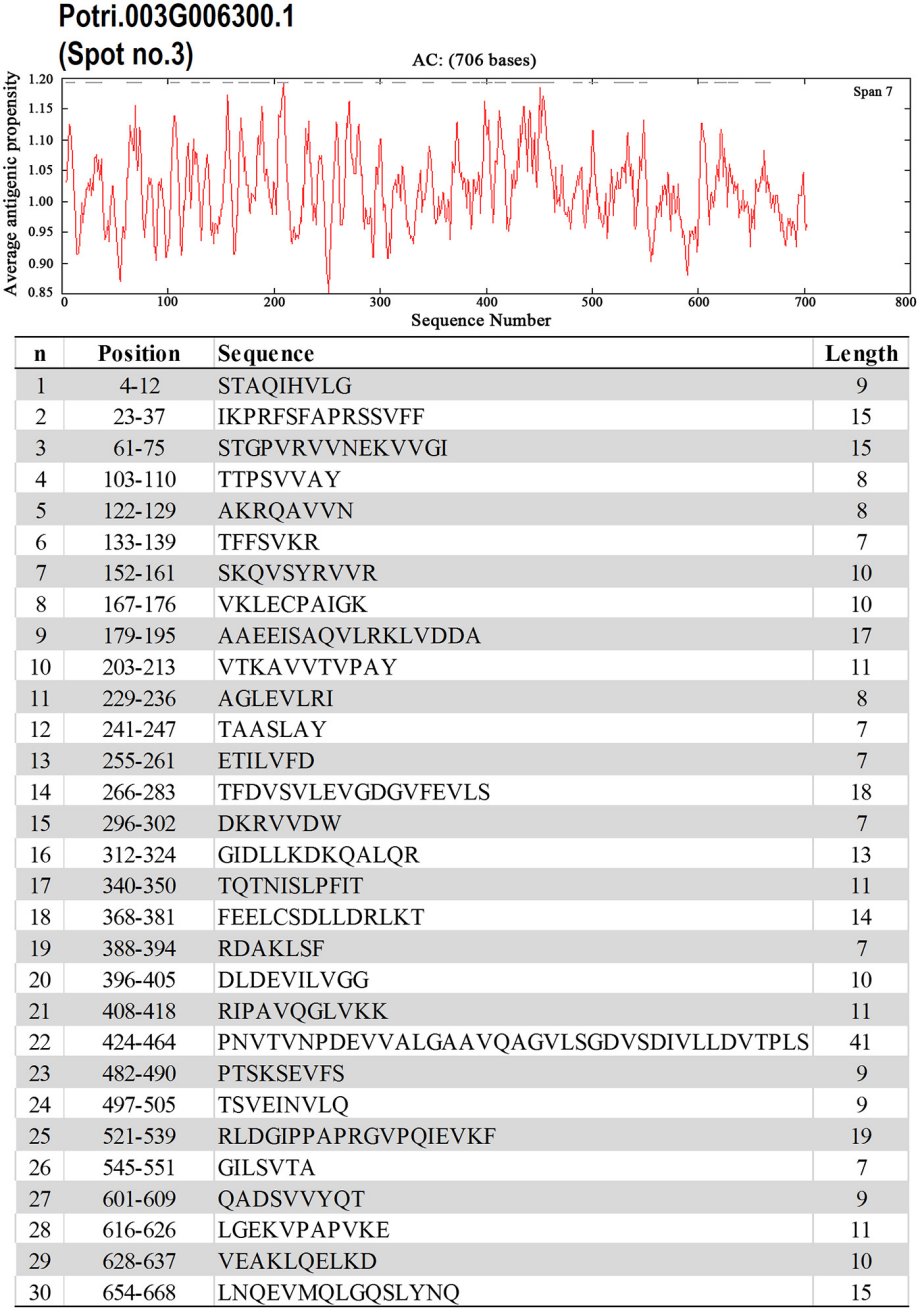
**One example (spot No. 3) of determined antigenic peptides in predicted antigen proteins**. Predictions are based on a table that reflects the occurrence of amino acid residues in experimentally known segmental epitopes (Kolaskar and Tongaonkar, 1990; Ivanciuc et al., 2003). Total prediction data of 28 candidate antigen proteins were shown in Figure S1.

To identify the likely allergen proteins present in *P. deltoides* mature pollen, the currently identified proteins were searched through SDAP, a web server that provides rapid, cross-referenced access to the sequences, structures and IgE epitopes of allergenic proteins (Ivanciuc et al., 2003). In this way, 28 mature pollen poplar proteins were predicted as being candidate allergens (Table 2). Then the potential antigenic peptides were determined using the method of Kolaskar and Tongaonkar (1990). Here, predictions are based on a table that reflects the occurrence of amino acid residues in experimentally known segmental epitopes. Segments are only reported if they have a minimum size of eight residues. From this search, the predicted antigenic peptides in the 28 predicted *P. deltoides* antigen proteins are shown in Figure 4 and Figure S1.

**Table 2:** **Predicted allergen related proteins in ***P. deltoides*** pollen**.

**Spot no**.	**Predicted allergens in *P. deltoids* pollen**				**Allergen**	**Corresponding of known allergen**			
	**Transcipt ID**	**Description**	**AA**	**Fragment of allergen**		**Accession No**.	**AA**	**Bit score**	**E score**
3	Potri.003G006300.1	Chloroplast heat shock protein 70-2	707	30	Cor a 10	CAC14168	668	322.5	5.00E-89
5	Potri.001G087500.1	Heat shock protein 70 (Hsp 70) family protein	667	27	Cor a 10	CAC14168	668	816.5	0.00E+00
12	Potri.001G285500.1	Mitochondrial HSO70 2	684	30	Cor a 10	CAC14168	668	400.4	1.70E-112
22	Potri.009G079700.1	Mitochondrial HSO70 2	683	31	Cor a 10	CAC14168	668	392.8	3.40E-110
44	Potri.006G116800.1	Enolase	446	20	Hev b 9	Q9LEI9	445	583.5	5.60E-168
51	Potri.015G131100.3	Enolase	443	18	Hev b 9	Q9LEJ0	445	545.9	1.20E-156
66	Potri.019G067200.1	Pectin lyase-like superfamily protein	394	18	Pla a 2	Q6H9K0	377	298.9	2.10E-82
78	Potri.002G034400.1	NmrA-like negative transcriptional regulator family protein	309	14	Bet v 6.0102	AAG22740	308	353.3	5.30E-99
80	Potri.012G114900.1	Pectin lyase-like superfamily protein	365	17	Sal k 1.0301	AAX11262	339	307.3	5.10E-85
84	Potri.010G117900.1	Aldolase superfamily protein	392	19	Sal s 3.0101	B5DGM7	363	278.9	2.00E-76
95	Potri.009G018600.1	Translation elongation factor EF1B	226	8	Pen c 24	AAR17475	228	122.6	8.40E-30
117	Potri.001G034400.1	Nascent polypeptide-associated complex, alpha subunit family protein	205	8	Hom s 2	CAA56869	215	124.7	1.60E-30
133	Potri.008G056300.2	Triosephosphate isomerase	255	10	Tri a 31.0101	CAC14917	253	339.2	6.40E-95
135	Potri.013G092600.1	Manganese superoxide dismutase 1	226	8	Pis v 4.0101	EF470980	230	324.5	1.40E-90
142	Potri.010G195700.1	HSP20-like superfamily protein	163	7	Cas s 9.0101	CAE46905	154	200.1	1.80E-53
144	Potri.001G254700.1	HSP20-like superfamily protein	143	7	Cas s 9.0101	CAE46905	154	131.4	7.70E-33
156	Potri.009G147900.1	HSP20-like superfamily protein	161	7	Cas s 9.0101	CAE46905	154	167.4	1.30E-43
158	Potri.018G083500.1	Thioredoxin-dependent peroxidase 1	163	8	Cand b 2	AAA34357	167	105.4	6.60E-25
161	Potri.001G392400.1	Pollen Ole e 1 allergen and extensin family protein	162	6	Lyc e LAT52	CAA33854	161	118.4	7.80E-29
164	Potri.011G111300.1	Pollen Ole e 1 allergen and extensin family protein	165	6	Lyc e LAT52	CAA33854	161	120.8	1.50E-29
181	Potri.007G018000.1	Thioredoxin H-type 1	123	4	Tri a 25.0101	Q9LDX4	125	114.8	5.50E-28
184	Potri.006G093500.1	HSP20-like superfamily protein	141	6	Cas s 9.0101	CAE46905	154	151.4	7.60E-39
197	Potri.009G146200.1	60S acidic ribosomal protein family	114	4	Pru du 5.0101	Q8H2B9	113	114.5	5.70E-28
200	Potri.003G047700.1	Profilin 3	132	7	Hev b 8.0201	Q9M7N0	131	195.6	2.90E-52
201	Potri.006G235200.2	Profilin 4	132	7	Pru du 4.0101	AY081850	131	202.6	2.20E-54
203	Potri.001G190800.2	Profilin 5	132	6	Lit c 1	AAL07320	131	194.4	6.60E-52
208	Potri.005G232700.2	Thioredoxin H-type 1	91	3	Zea m 25.0101	Q4W1F7	128	89.4	1.80E-20
216	Potri.018G057600.7	Profilin 4	93	5	Hev b 8.0204	Q9LEI8	131	146.3	1.40E-37

In *P. deltoids* pollen, four small Hsps (spots No. 142, 144, 156, and 184) and four Hsp70 (spots No. 3, 5, 12, and 22) were identified as corresponding to allergenic molecules (Table 2). It has reported that class I small heat shock protein (Hsp) is one of allergens in soybean (Gagnon et al., 2010). Hsp70 proteins have been demonstrated to bind to human IgE from patients sensitized to penicillium (Shen et al., 1997), cystic echinococcosis (Ortona et al., 2003), and to corn and wheat dust (Chiung et al., 2000). Spots No. 161 and 164 correspond to pollen Ole e 1 allergen, which was first purified from *Olea europea* (Lauzurica et al., 1988) and named as Ole e 1 according to the IUIS nomenclature (Marsh et al., 1986). This protein is surmised to control pregermination and pollen tube emergence and guidance (de Dios Alché et al., 2004). In addition, thioredoxin proteins (spots No. 181 and 208) and profilin (spots No. 200, 201, 203, and 216) were identified in our study, consistent with the finding that these two types of proteins have previously been reported as being allergens in wheat, maize, and other plant species (Kleber-Janke et al., 1999; Weichel et al., 2006; Villalta and Asero, 2010).

### Expression patterns of the predicted allergen genes across various tissues

**Figure 5:**
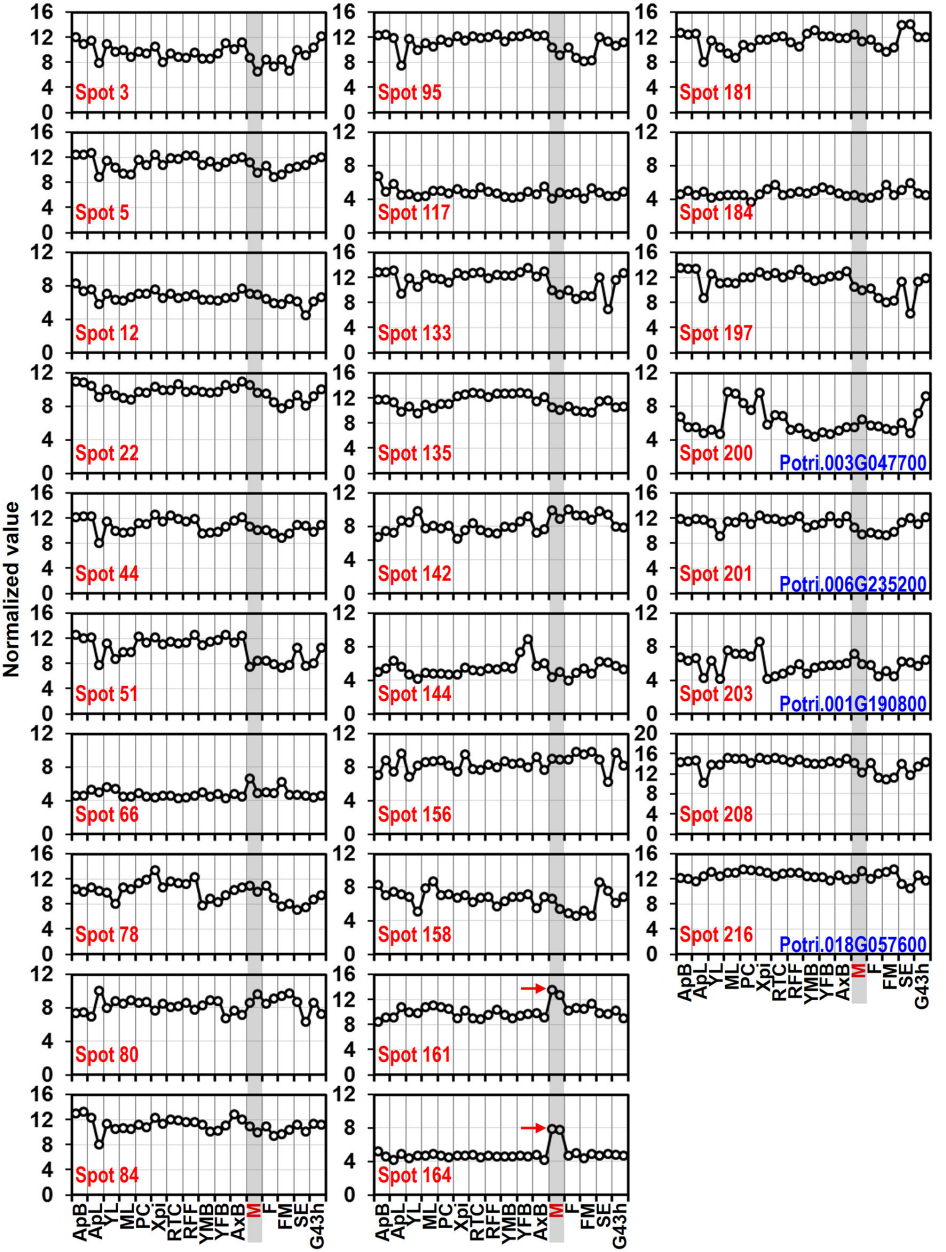
**Expression profiles of genes coding 28 predicted allergen related proteins across different tissues**. The Affymetrix microarray data were obtained from NCBI Gene Expression Omnibus (GEO) database under the series accession number GSE21481. ApB, Shoot apex; ApL, Young leaves at apex; YL, Young leaves plastochron #2; ML, Mature leaves plastochron #5; PC, Phloem and Cortex; XPi, Developing xylem and pith; RTC, Roots from tissue culture; RFF, Roots from field trees; YMB, Male floral bud initials; YFB, Female floral bud initials; AxB, Axillary buds; M, Male catkin - 3 stages pooled; F, Female catkin post-pollination; FM, Mature catkin before seed release; SE, Seed; G43 h, Seedling 43 h post-imbibition. The spots No. 200, 201, 203, and 216 correspond to profilin (see Table 1).

Whole genome microarray proved to be a useful means for studying gene expression profiles in poplar (Zhang et al., 2013). To examine whether the predicted pollen-allergen genes presently characterized are expressed in poplar and to study their expression patterns, a comprehensive analysis was conducted based on an Affymetrix microarray data (GSE21481). The expression patterns of the 28 predicted poplar allergen genes across various tissues are shown in Figure 5. Noticeably, two genes corresponding to spots No. 161 and 164 (pollen Ole e 1 allergen proteins) were highly expressed in male catkin, thereby suggesting their specific expression in pollen. It is known that *Ole e* I is expressed in pollen wall and tapetum but not in petals, roots, or leaves (de Dios Alché et al., 1999). Muschietti et al. (1994) reported that antisense repression of *LAT52*, a homolog of *Ole e* I, was associated with abnormal pollen function, consistent with a role of this protein in pollen hydration and/or pollen germination. While only three (spots No. 66, 161, and 164) of pollen allergen genes were highly expressed in male catkin. These genes might play important roles in not only reproductive but also vegetative development. Thus, our data contribute to the identification of new pollen allergenic genes.

**Figure 6:**
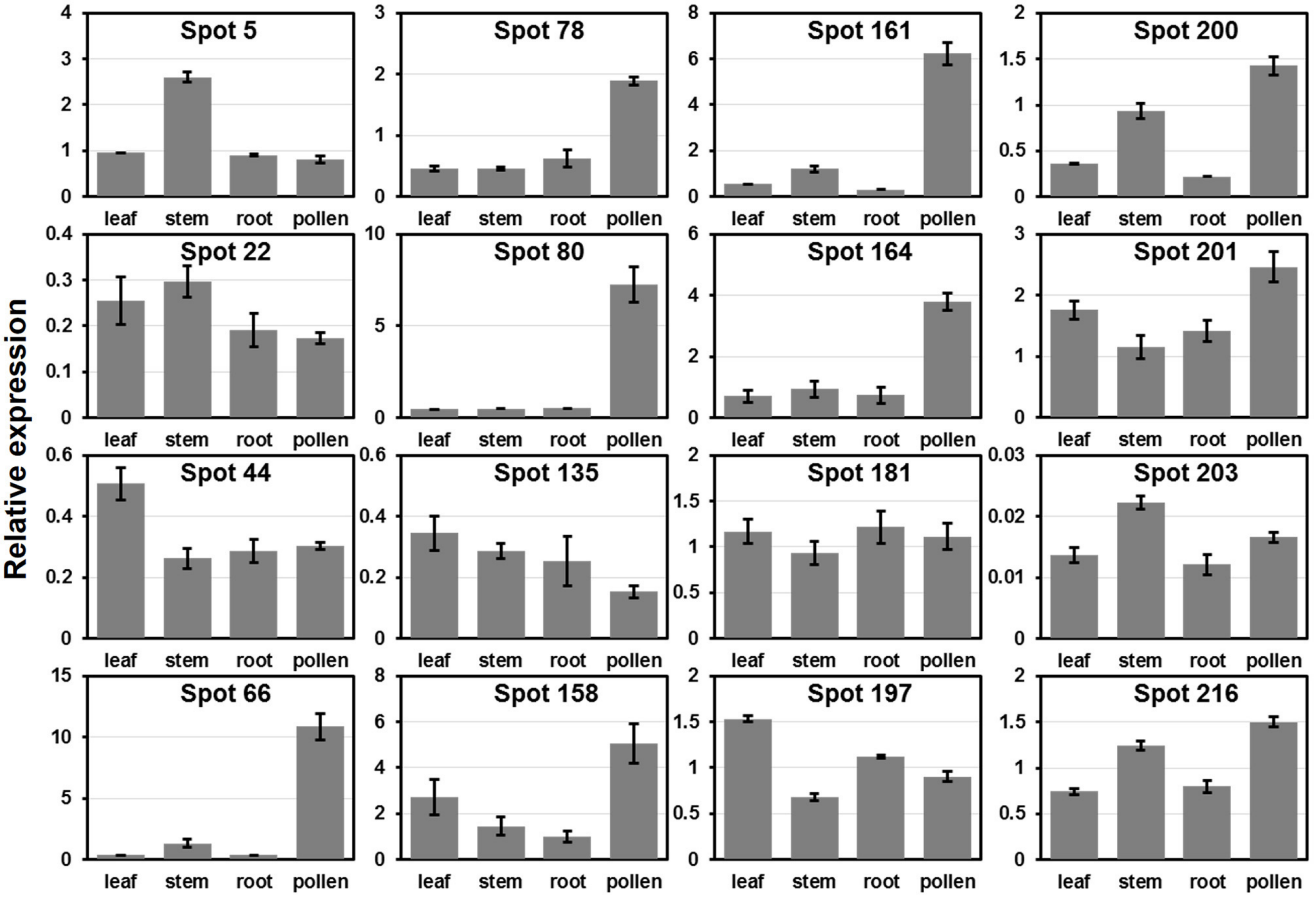
**Expression analysis of allergen related genes in different tissues using qRT-PCR**. The relative mRNA abundance of 16 allergen related genes presently described were quantified in four tissues (leaf, stem, root, and pollen). The average expression of each gene was calculated relatively to the reference gene PtActin. Relative expression represents log2 expression values. All primer sequences are listed in Table S1. The spots No. 200, 201, 203, and 216 correspond to identified profilin (see Table 1).

To verify the expression patterns of the presently characterized predicted allergen genes, qRT-PCR analysis was performed on four tissues for 16 genes (Figure 6). Microarray data in Figure 5 show that the genes corresponding to spot No. 66 (pectin lyase-like family), spot No. 80 (pectin lyase-like family), spot No. 161 (extension family), and spot No. 164 (extension family) were highly expressed in male catkin. Our present qRT-PCR results indicate that the four genes were high expressed in pollen (Figure 6). Moreover, two genes corresponding to spot No. 78 (NmrA-like negative transcriptional regulator family protein) and spot No. 158 (thioredoxin-dependent peroxidase) also exhibited high abundance in pollen. One likely reason is the pollen we used in qRT-PCR was purer than the male catkin used in the microarray analysis in tissue level. In general, the present qRT-PCR results were in good agreement with the microarray data sets analyzed in this study.

### Profilin gene family and its expression patterns in poplar

Profilin was first identified as an allergen in birch pollen Bet v2 (Valenta et al., 1991). These proteins probably function as important mediators of membrane-cytoskeleton communication (Machesky and Poland, 1993). Profilins specifically bind to several ligands, that is, actin, phosphatidylinositol-4,5-bisphosphate (PIP2), and poly-L-proline. These characteristics enable them to participate in the regulation of actin polymerization and to interact with the PIP2 pathway of signal transduction (Vieths et al., 2002). Plant profilins exhibit conserved amino acid sequences, share IgE-reactive epitopes, and correspond to highly cross-reactive allergens (Sankian et al., 2005). In the present study, four profilin proteins were identified in *P. deltoides* mature pollen (Tables 1, 2). To identify all profilin genes in poplar, we performed a BLASTp search against *P. trichocarpa* genome using profilin protein sequences in *Arabidopsis* and rice as queries. After confirmation of the protein secondary structure, a total of four profilin genes in poplar were identified. It is noted that all members of this poplar profilin gene family were identified in our proteomic study, suggesting that these profilin proteins are present in high abundance in poplar pollen.

**Figure 7:**
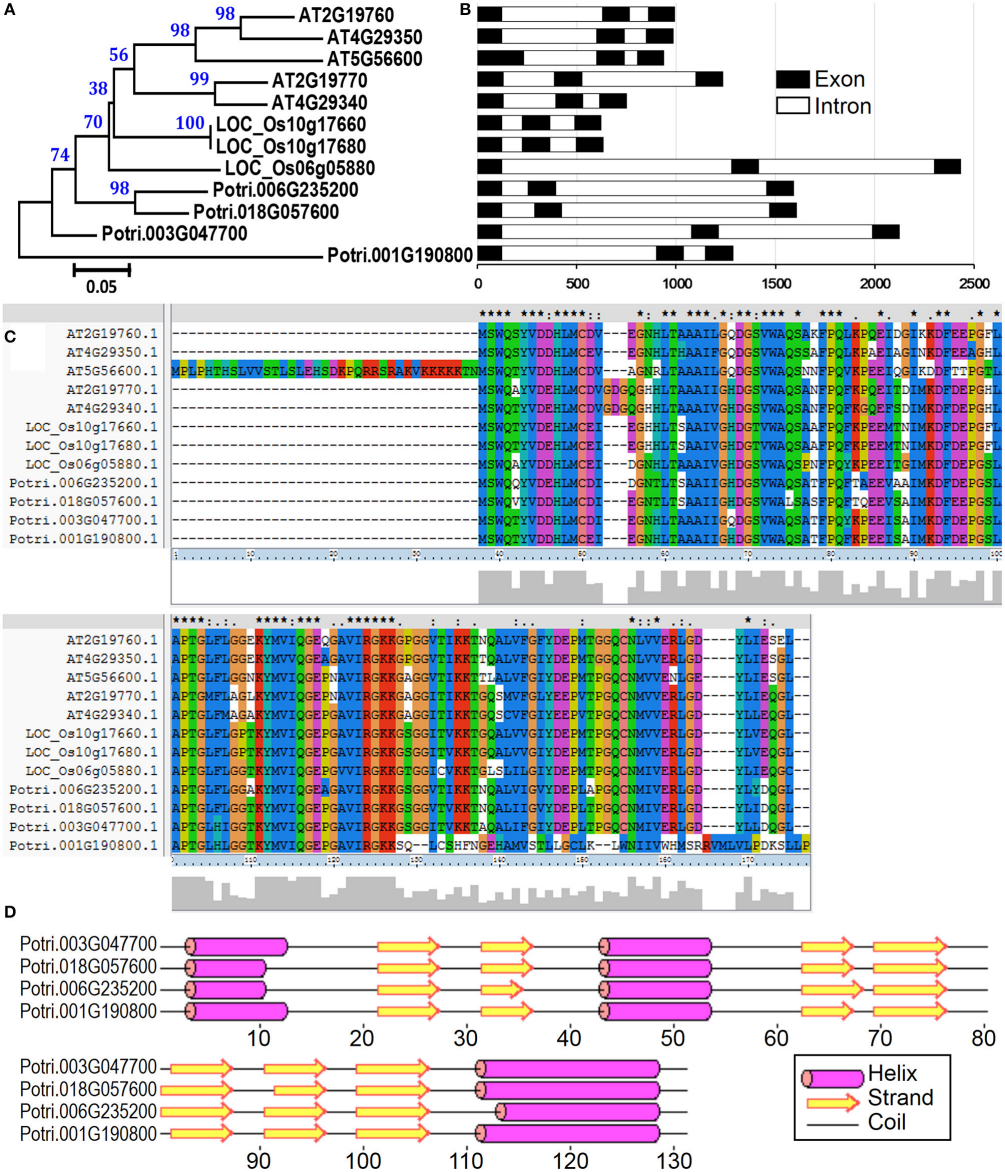
**The profilin gene family from poplar, *Arabidopsis*, and rice**. **(A)** A multiple alignment of full-length profilin protein sequences from three species was executed using Clustal X2.1 and a phylogenetic tree was constructed by the neighbor-joining (NJ) method with 1000 bootstrap replicates. Bootstrap support values are shown on each node. **(B)** Gene structures of profilin genes from three species. Exons and introns are represented with black and white boxes. **(C)** Amino acid sequence alignment of profilin proteins in three species. **(D)** Secondary structure prediction of profilin proteins in poplar.

To examine the evolutionary relationships of profilin proteins, we constructed a phylogenetic tree by neighbor-joining method using the full-length profilin proteins in poplar, *Arabidopsis*, and rice. The phylogenetic tree and amino acid sequences alignment indicate that profilin proteins were highly conserved across three species (Figures 7A,C). We then analyzed the gene structure in the coding sequence of the profilin gene family. The profilin genes in the three species have three exons with conserved length, except one *Arabidopsis* profilin gene (At5g56600) of which first exon is longer than for the others (Figure 7B). Similar to known profilin structures, poplar profilins consist of three helices and seven β-strands (Figure 7D). In birch pollen profilin, the seven β-strands appear as two orthogonal β-sheets, the first sheet being formed by β1, β2, β4, β5, and β6 strands, while the second sheet is formed by β3 and β7 strands (Fedorov et al., 1997).

We then compared the expression patterns of profilin genes in poplar, *Arabidopsis*, and rice. As shown in Figure 6, two poplar genes (corresponding to spots No. 200 and 201) out of the four poplar profilin genes (corresponding to spots No. 200, 201, 203, and 216) exhibit high expression level in pollen. In *Arabidopsis*, two profilins (At2g19770 and At4g29340) are highly expressed in flowers and floral organs (Figure S2A). In contrast in rice only one profilin (LOC_Os19g17680) is highly expressed in anther (Figure S2B). The similar expression patterns of these genes across plant species (poplar, *Arabidopsis*, and rice, Figure 6 and Figure S2) suggest that they kept some conserved functions during plant evolution.

## Conclusion

In conclusion, the present study analyzed the proteome of *P. deltoides* mature pollen for the first time. A total of 403 protein spots were isolated by 2-DE, and 178 distinct proteins were identified from 218 protein spots using MALDI-TOF/TOF MS/MS analysis. Furthermore, 28 proteins were identified as putative allergens and their expression patterns across various tissues were analyzed. The expression patterns across various tissues showed that several of allergenic genes are highly expressed in pollen. Moreover, the members of profilin allergen family were analyzed and their expression patterns were compared with the homologous in *Arabidopsis* and rice. The similar expression profiles of profilins across different plant species support their conserved functions during plant evolution.

### Conflict of interest statement

The authors declare that the research was conducted in the absence of any commercial or financial relationships that could be construed as a potential conflict of interest.

## Supplementary material

Figure S1 **The determined antigenic peptides in 28 predicted antigen proteins**. Predictions are based on a table that reflects the occurrence of amino acid residues in experimentally known segmental epitopes (Kolaskar and Tongaonkar, 1990; Ivanciuc et al., 2003).

Figure S2 **Expression patterns of Profilin genes in various tissues of Arabidopsis (A) and rice (B)**. The microarray data was obtained from AtGenExpress Visualization Tool (AVT, http://jsp.weigelworld.org/expviz/) for Arabidopsis and the Rice Expression Profile Database (RiceXPro, http://ricexpro.dna.affrc.go.jp/) for rice.

Table S1 **Sequences of qRT-PCR primers**.

## References

[B1] Aalberse R. C. (2000). Structural biology of allergens. J. Allergy Clin. Immunol. 106, 228–238. 10.1067/mai.2000.108434 10932064

[B2] Akagawa M. Handoyo T. Ishii T. Kumazawa S. Morita N. Suyama K. (2007). Proteomic analysis of wheat flour allergens. J. Agric. Food Chem. 55, 6863–6870. 10.1021/jf070843a 17655322

[B3] Ashburner M. Ball C. A. Blake J. A. Botstein D. Butler H. Cherry J. M.. (2000). Gene Ontology: tool for the unification of biology. Nat. Genet. 25, 25–29. 10.1038/75556 10802651 PMC3037419

[B4] Bartra J. Sastre J. Del Cuvillo A. Montoro J. Jauregui I. Davila I.. (2009). From pollinosis to digestive allergy. J. Investig. Allergol. Clin. Immunol. 19(Suppl. 1), 3–10. 19476048

[B5] Becker J. D. Boavida L. C. Carneiro J. Haury M. Feijó J. A. (2003). Transcriptional profiling of *Arabidopsis* tissues reveals the unique characteristics of the pollen transcriptome. Plant Physiol. 133, 713–725. 10.1104/pp.103.028241 14500793 PMC219046

[B6] Bohle B. (2004). T lymphocytes and food allergy. Mol. Nutr. Food Res. 48, 424–433. 10.1002/mnfr.200400003 15508177

[B7] Breiteneder H. Ebner C. (2000). Molecular and biochemical classification of plant-derived food allergens. J. Allergy Clin. Immunol. 106, 27–36. 10.1067/mai.2000.106929 10887301

[B8] Brett D. Pospisil H. Valcárcel J. Reich J. Bork P. (2002). Alternative splicing and genome complexity. Nat. Genet. 30, 29–30. 10.1038/ng803 11743582

[B9] Canovas F. M. Dumas-Gaudot E. Recorbet G. Jorrin J. Mock H. P. Rossignol M. (2004). Plant proteome analysis. Proteomics 4, 285–298. 10.1002/pmic.200300602 14760698

[B10] Chiung Y. M. Lin B. L. Yeh C. H. Lin C. Y. (2000). Heat shock protein (hsp 70) -related epitopes are common allergenic determinants for barley and corn antigens. Electrophoresis 21, 297–300. 10.1002/(SICI)1522-2683(20000101)21:2<297::AID-ELPS297>3.0.CO;2-U 10675004

[B11] Conesa A. Götz S. García-Gómez J. M. Terol J. Talón M. Robles M. (2005). Blast2GO: a universal tool for annotation, visualization and analysis in functional genomics research. Bioinformatics 21, 3674–3676. 10.1093/bioinformatics/bti610 16081474

[B12] de Dios Alché J. Castro A. J. Olmedilla A. Fernandez M. D. C. Rodríguez R. Villalba M.. (1999). The major olive pollen allergen (Ole e I) shows both gametophytic and sporophytic expression during anther development, and its synthesis and storage takes place in the RER. J. Cell Sci. 112, 2501–2509. 10393806 10.1242/jcs.112.15.2501

[B13] de Dios Alché J. M'rani-Alaoui M. Castro A. J. Rodríguez-García M. I. (2004). Ole e 1, the major allergen from olive (*Olea europaea* L.) pollen, increases its expression and is released to the culture medium during *in vitro* germination. Plant Cell Physiol. 45, 1149–1157. 10.1093/pcp/pch127 15509837

[B14] Fedorov A. A. Ball T. Mahoney N. M. Valenta R. Almo S. C. (1997). The molecular basis for allergen cross-reactivity: crystal structure and IgE-epitope mapping of birch pollen profilin. Structure 5, 33–45. 10.1016/S0969-2126(97)00164-0 9016715

[B15] Gagnon C. Poysa V. Cober E. R. Gleddie S. (2010). Soybean allergens affecting North American patients identified by 2D gels and mass spectrometry. Food Anal. Method 3, 363–374. 10.1007/s12161-009-9090-3

[B16] Galland M. Huguet R. Arc E. Cueff G. Job D. Rajjou L. (2014). Dynamic proteomics emphasizes the importance of selective mRNA translation and protein turnover during Arabidopsis seed germination. Mol. Cell Proteomics 13, 252–268. 10.1074/mcp.M113.032227 24198433 PMC3879618

[B17] Hirano H. Islam N. Kawasaki H. (2004). Technical aspects of functional proteomics in plants. Phytochemistry 65, 1487–1498. 10.1016/j.phytochem.2004.05.019 15276446

[B18] Holmes-Davis R. Tanaka C. K. Vensel W. H. Hurkman W. J. Mccormick S. (2005). Proteome mapping of mature pollen of *Arabidopsis thaliana*. Proteomics 5, 4864–4884. 10.1002/pmic.200402011 16247729

[B19] Honys D. Twell D. (2003). Comparative analysis of the *Arabidopsis* pollen transcriptome. Plant Physiol. 132, 640–652. 10.1104/pp.103.020925 12805594 PMC167004

[B20] Ipsen H. Løwenstein H. (1997). Basic features of crossreactivity in tree and grass pollen allergy. Clin. Rev. Allergy Immunol. 15, 389–396. 10.1007/BF02737734 9484576

[B21] Ivanciuc O. Schein C. H. Braun W. (2003). SDAP: database and computational tools for allergenic proteins. Nucleic Acids Res. 31, 359–362. 10.1093/nar/gkg010 12520022 PMC165457

[B22] Jensen O. N. (2004). Modification-specific proteomics: characterization of post-translational modifications by mass spectrometry. Curr. Opin. Chem. Biol. 8, 33–41. 10.1016/j.cbpa.2003.12.009 15036154

[B23] Kitta K. Ohnishi-Kameyama M. Moriyama T. Ogawa T. Kawamoto S. (2006). Detection of low-molecular weight allergens resolved on two-dimensional electrophoresis with acid-urea polyacrylamide gel. Anal. Biochem. 351, 290–297. 10.1016/j.ab.2005.12.030 16457770

[B24] Kleber-Janke T. Crameri R. Appenzeller U. Schlaak M. Becker W.-M. (1999). Selective cloning of peanut allergens, including profilin and 2S albumins, by phage display technology. Int. Arch. Allergy Immunol. 119, 265–274. 10.1159/000024203 10474031

[B25] Kolaskar A. Tongaonkar P. C. (1990). A semi-empirical method for prediction of antigenic determinants on protein antigens. FEBS Lett. 276, 172–174. 10.1016/0014-5793(90)80535-Q 1702393

[B26] Larkin M. A. Blackshields G. Brown N. P. Chenna R. Mcgettigan P. A. Mcwilliam H.. (2007). Clustal W and Clustal X version 2.0. Bioinformatics 23, 2947–2948. 10.1093/bioinformatics/btm404 17846036

[B27] Lauzurica P. Gurbindo C. Maruri N. Galocha B. Diaz R. González J.. (1988). Olive (*Olea europea*) pollen allergens-I immunochemical characterization by immunoblotting, crie and immunodetection by a monoclonal antibody. Mol. Immunol. 25, 329–335. 10.1016/0161-5890(88)90027-2 3398857

[B28] Loraine A. E. McCormick S. Estrada A. Patel K. Qin P. (2013). RNA-seq of *Arabidopsis* pollen uncovers novel transcription and alternative splicing. Plant Physiol. 162, 1092–1109. 10.1104/pp.112.211441 23590974 PMC3668042

[B29] Ma H. (2005). Molecular genetic analyses of microsporogenesis and microgametogenesis in flowering plants. Annu. Rev. Plant Biol. 56, 393–434. 10.1146/annurev.arplant.55.031903.141717 15862102

[B30] Machesky L. M. Poland T. D. (1993). Profilin as a potential mediator of membrane-cytoskeleton communication. Trends Cell Biol. 3, 381–385. 10.1016/0962-8924(93)90087-H 14731655

[B31] Marsh D. Goodfriend L. King T. Lowenstein H. Platts-Mills T. (1986). Allergen nomenclature. Bull. World Health Organ. 64, 767. 3492310 PMC2490960

[B32] Mccormick S. (2004). Control of male gametophyte development. Plant Cell 16, S142–S153. 10.1105/tpc.016659 15037731 PMC2643393

[B33] Mohapatra S. S. Knox R. B. (1996). Pollen Biotechnology, Gene Expression and Allergen Characterization. London: Chapman & Hall.

[B34] Muschietti J. Dircks L. Vancanneyt G. Mccormick S. (1994). LAT52 protein is essential for tomato pollen development: pollen expressing antisense LAT52 RNA hydrates and germinates abnormally and cannot achieve fertilization. Plant J. 6, 321–338. 10.1046/j.1365-313X.1994.06030321.x 7920720

[B35] Nakamura R. Teshima R. (2013). Proteomics-based allergen analysis in plants. J. Proteomics 93, 40–49. 10.1016/j.jprot.2013.03.018 23568023

[B36] Noir S. Bräutigam A. Colby T. Schmidt J. Panstruga R. (2005). A reference map of the *Arabidopsis thaliana* mature pollen proteome. Biochem. Biophys. Res. Commun. 337, 1257–1266. 10.1016/j.bbrc.2005.09.185 16242667

[B37] Ortona E. Margutti P. Delunardo F. Vaccari S. Rigano R. Profumo E.. (2003). Molecular and immunological characterization of the C-terminal region of a new *Echinococcus granulosus* heat shock protein 70. Parasite Immunol. 25, 119–126. 10.1046/j.1365-3024.2003.00617.x 12911519

[B38] Picariello G. Mamone G. Addeo F. Ferranti P. (2011). The frontiers of mass spectrometry-based techniques in food allergenomics. J. Chromatogr. A 1218, 7386–7398. 10.1016/j.chroma.2011.06.033 21737089

[B39] Pina C. Pinto F. Feijó J. A. Becker J. D. (2005). Gene family analysis of the *Arabidopsis* pollen transcriptome reveals biological implications for cell growth, division control, and gene expression regulation. Plant Physiol. 138, 744–756. 10.1104/pp.104.057935 15908605 PMC1150393

[B40] Rajjou L. Gallardo K. Debeaujon I. Vandekerckhove J. Job C. Job D. (2004). The effect of α-amanitin on the Arabidopsis seed proteome highlights the distinct roles of stored and neosynthesized mRNAs during germination. Plant Physiol. 134, 1598–1613. 10.1104/pp.103.036293 15047896 PMC419834

[B41] Sankian M. Varasteh A. Pazouki N. Mahmoudi M. (2005). Sequence homology: a poor predictive value for profilins cross-reactivity. Clin. Mol. Allergy 3, 13. 10.1186/1476-7961-3-13 16153305 PMC1253521

[B42] Shahali Y. Sutra J. P. Haddad I. Vinh J. Guilloux L. Peltre G.. (2012). Proteomics of cypress pollen allergens using double and triple one-dimensional electrophoresis. Electrophoresis 33, 462–469. 10.1002/elps.201100324 22287175

[B43] Shen H. D. Au L. C. Lin W. L. Liaw S. F. Tsai J. J. Han S. H. (1997). Molecular cloning and expression of a *Penicillium citrinum* allergen with sequence homology and antigenic crossreactivity to a hsp 70 human heat shock protein. Clin. Exp. Allergy 27, 682–690. 10.1111/j.1365-2222.1997.tb01197.x 9208190

[B44] Sheng X. Hu Z. Lü H. Wang X. Baluška F. Šamaj J. Lin J. (2006). Roles of the ubiquitin/proteasome pathway in pollen tube growth with emphasis on MG132-induced alterations in ultrastructure, cytoskeleton, and cell wall components. Plant Physiol. 141, 1578–1590. 10.1104/pp.106.081703 16778013 PMC1533934

[B45] Sheoran I. S. Ross A. R. Olson D. J. Sawhney V. K. (2007). Proteomic analysis of tomato (*Lycopersicon esculentum*) pollen. J. Exp. Bot. 58, 3525–3535. 10.1093/jxb/erm199 17921476

[B46] Sheoran I. S. Sproule K. A. Olson D. J. Ross A. R. Sawhney V. K. (2006). Proteome profile and functional classification of proteins in *Arabidopsis thaliana* (Landsberg erecta) mature pollen. Sex. Plant Reprod. 19, 185–196. 10.1007/s00497-006-0035-3

[B47] Sicherer S. H. (2001). Clinical implications of cross-reactive food allergens. J. Allergy Clin. Immunol. 108, 881–890. 10.1067/mai.2001.118515 11742262

[B48] Singh A. B. Shahi S. (2008). Aeroallergens in clinical practice of allergy in India-ARIA Asia Pacific Workshop report. Asian Pac. J. Allergy Immunol. 26, 245–256. 19317344

[B49] Sircar G. Chakrabarti H. S. Saha B. Gupta-Bhattacharya S. (2012). Identification of aero-allergens from *Rhizopus oryzae*: an immunoproteomic approach. J. Proteomics 77, 455–468. 10.1016/j.jprot.2012.09.023 23041133

[B50] Supek F. Bosnjak M. Skunca N. Smuc T. (2011). REVIGO summarizes and visualizes long lists of gene ontology terms. PLoS ONE 6:e21800. 10.1371/journal.pone.0021800 21789182 PMC3138752

[B51] Tamura K. Peterson D. Peterson N. Stecher G. Nei M. Kumar S. (2011). MEGA5: molecular evolutionary genetics analysis using maximum likelihood, evolutionary distance, and maximum parsimony methods. Mol. Biol. Evol. 28, 2731–2739. 10.1093/molbev/msr121 21546353 PMC3203626

[B52] Taylor G. (2002). Populus: Arabidopsis for forestry. Do we need a model tree? Ann. Bot. 90, 681–689. 10.1093/aob/mcf255 12451023 PMC4240366

[B53] Valenta R. Duchene M. Pettenburger K. Sillaber C. Valent P. Bettelheim P.. (1991). Identification of profilin as a novel pollen allergen; IgE autoreactivity in sensitized individuals. Science 253, 557–560. 10.1126/science.1857985 1857985

[B54] Vieths S. Scheurer S. Ballmer-Weber B. (2002). Current understanding of cross−reactivity of food allergens and pollen. Ann. N.Y. Acad. Sci. 964, 47–68. 10.1111/j.1749-6632.2002.tb04132.x 12023194

[B55] Villalta D. Asero R. (2010). Sensitization to the pollen pan-allergen profilin. Is the detection of immunoglobulin E to multiple homologous proteins from different sources clinically useful? J. Invest. Allergy Clin. 20, 591. 21314000

[B56] Volkov R. A. Panchuk I. I. Schöffl F. (2005). Small heat shock proteins are differentially regulated during pollen development and following heat stress in tobacco. Plant Mol. Biol. 57, 487–502. 10.1007/s11103-005-0339-y 15821976

[B57] Weichel M. Glaser A. G. Ballmer-Weber B. K. Schmid-Grendelmeier P. Crameri R. (2006). Wheat and maize thioredoxins: a novel cross-reactive cereal allergen family related to baker's asthma. J. Allergy Clin. Immunol. 117, 676–681. 10.1016/j.jaci.2005.11.040 16522470

[B58] Wilkins M. R. Pasquali C. Appel R. D. Ou K. Golaz O. Sanchez J.-C.. (1996). From proteins to proteomes: large scale protein identification by two dimensional electrophoresis and amino acid analysis. Biotechnolgy 14, 61–65. 10.1038/nbt0196-61 9636313

[B59] Yano H. Kuroda S. (2008). Introduction of the disulfide proteome: application of a technique for the analysis of plant storage proteins as well as allergens. J. Proteome Res. 7, 3071–3079. 10.1021/pr8003453 18624400

[B60] Zhang J. Li J. Liu B. Zhang L. Chen J. Lu M. (2013). Genome-wide analysis of the *Populus* Hsp90 gene family reveals differential expression patterns, localization, and heat stress responses. BMC Genomics 14:532. 10.1186/1471-2164-14-532 23915275 PMC3750472

[B61] Zou J. Song L. Zhang W. Wang Y. Ruan S. Wu W. H. (2009). Comparative proteomic analysis of *Arabidopsis* mature pollen and germinated pollen. J. Integr. Plant Biol. 51, 438–455. 10.1111/j.1744-7909.2009.00823.x 19508356

