# Supplementary material for: Proteomic analysis and candidate allergenic proteins in Populus deltoides CL. “2KEN8” mature pollen
Source: Front Plant Sci. 2015 Jul 29;6:548. doi: 10.3389/fpls.2015.00548 (PMC4518142; doi:10.3389/fpls.2015.00548)
Supplement: Figure S1 — The determined antigenic peptides in 28 predicted antigen proteins. Predictions are based on a table that reflects the occurrence of amino acid residues in experimentally known segmental epitopes (Kolaskar and Tongaonkar, 1990; Ivanciuc et al., 2003). [file Image1.PDF]

Potri.003G006300.1 (Spot no.3)

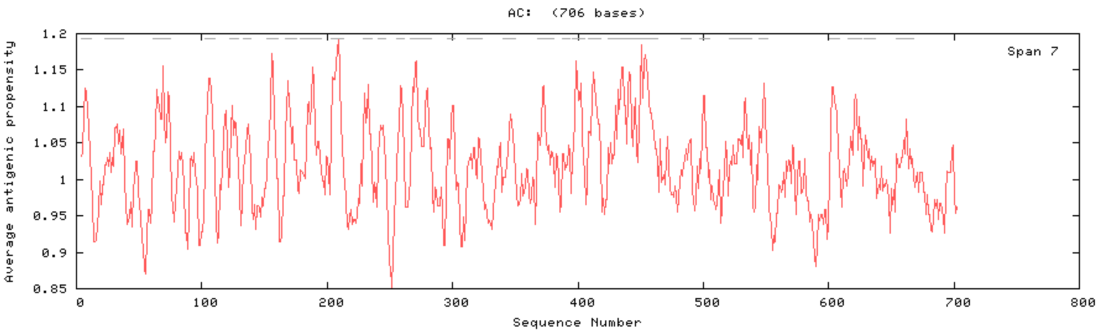

n	Position	Sequence	Length
1	4-12	STAQIHVLG	9
2	23-37	IKPRFSFAPRSSVFF	15
3	61-75	STGPVRVVNEKVVGI	15
4	103-110	TTPSVVAY	8
5	122-129	AKRQAVVN	8
6	133-139	TFFSVKR	7
7	152-161	SKQVSYRVVR	10
8	167-176	VKLECPAIGK	10
9	179-195	AAEEISAQVLRKLVDDA	17
10	203-213	VTKAVVTVPAY	11
11	229-236	AGLEVLRI	8
12	241-247	TAASLAY	7
13	255-261	ETILVFD	7
14	266-283	TFDVSVLEVGDGVFEVLS	18
15	296-302	DKRVVDW	7
16	312-324	GIDLLKDKQALQR	13
17	340-350	TQTNISLPFIT	11
18	368-381	FEELCSDLLDRLKT	14
19	388-394	RDAKLSF	7
20	396-405	DLDEVILVGG	10
21	408-418	RIPAVQGLVKK	11
22	424-464	PNVTVNPDEVVALGAAVQAGVLSGDVSDIVLLDVTPLS	41
23	482-490	PTSKSEVFS	9
24	497-505	TSVEINVLQ	9
25	521-539	RLDGIPPAPRGVPQIEVKF	19
26	545-551	GILSVTA	7
27	601-609	QADSVVYQT	9
28	616-626	LGEKVPAPVKE	11
29	628-637	VEAKLQELKD	10
30	654-668	LNQEVMQLGQSLYNQ	15

Fig. S1

Potri.001G087500.1 (Spot no.5)

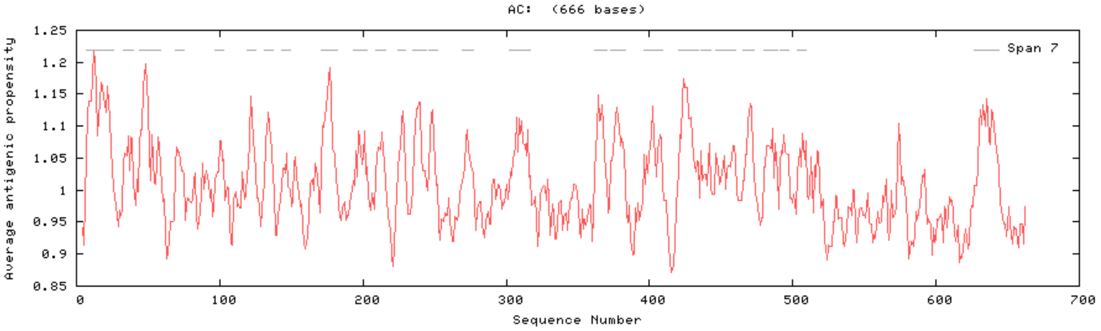

n	Position	Sequence	Length
1	7-26	ARGVVVSAIILFGCLFAISI	20
2	33-40	KLGTVIGI	8
3	44-59	TTYSCVGVYKNGHVEI	16
4	69-75	TPSWVAF	7
5	97-103	TIFDVKR	7
6	119-125	KLFPYKI	7
7	131-137	KPYIQVK	7
8	143-149	TKVFSPE	7
9	171-182	IKDAVVTVPAYF	12
10	193-203	AGIIAGLNVAR	11
11	209-216	TAAAIAYG	8
12	224-230	KNILVFD	7
13	235-244	TFDVSVLTID	10
14	246-252	GVFEVLS	7
15	269-277	MEYFIKLIK	9
16	302-317	KRALSSQHQVRVEIES	16
17	362-370	IDEIVLVGG	9
18	373-383	RIPKVQQLLKD	11
19	396-409	PDEAVAFGAAVQGG	14
20	420-434	KDILLLDVAPLTLGI	15
21	436-443	TVGGVMTK	8
22	446-460	PRNTVIPTKKSQVFT	15
23	465-473	QQTTVSIQV	9
24	480-489	LTKDCRLLGK	10
25	491-497	DLTGVPP	7
26	503-509	PQIEVTF	7
27	627-644	KLKEVEAVCNPIITAVYQ	18

Fig. S1

Potri.001G285500.1 (Spot no.12)

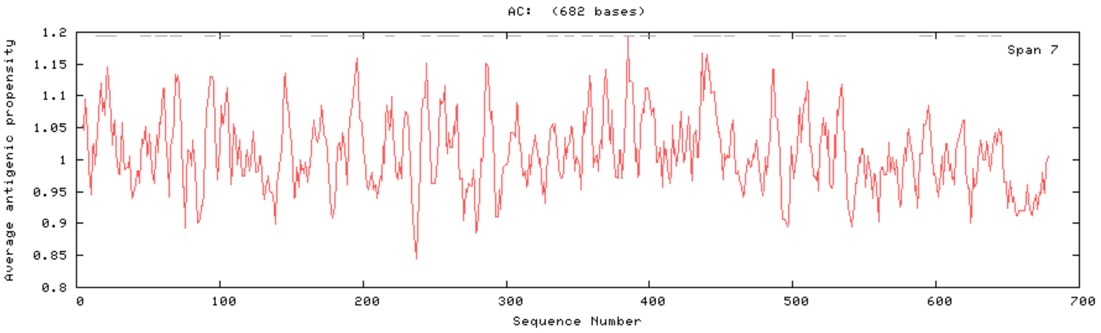

n	Position	Sequence	Length
1	14-28	DVASAPLSAYRCLTN	15
2	45-52	GLSRAFSA	8
3	55-63	AGGDVIGVD	9
4	66-73	TTNSCVAV	8
5	90-97	TTPSVVAF	8
6	100-107	KGELLVGT	8
7	142-150	KMVPYKIVR	9
8	164-175	QYSPSQIGAFIL	12
9	190-201	ITKAVITVPAYF	12
10	216-222	AGLDVQR	7
11	241-247	GLIAVFD	7
12	252-267	TFDVSILEISNGVFEV	16
13	284-291	TLLEYLVD	8
14	301-310	LSKDKLALQR	10
15	329-336	EINLPFIT	8
16	341-347	GAKHLNI	7
17	353-360	KFESLVNH	8
18	365-375	TRIPCKNCLKD	11
19	382-389	EVDEVLLV	8
20	394-404	RVPKVQDIVAE	11
21	431-450	LRGDVKELLLLDVTPLSLGI	20
22	452-459	TLGGIFTR	8
23	483-491	TQVGIKVLQ	9
24	502-513	LLGEFDLVGIPP	12
25	519-525	PQIEVTF	7
26	529-537	ANGIVTVSA	9
27	589-597	DTTIYSVEK	9
28	614-620	IEDAVAD	7
29	629-636	SVDDIKSK	8
30	639-646	AANKAVSK	8

Fig. S1

Potri.009G079700.1 (Spot no.22)

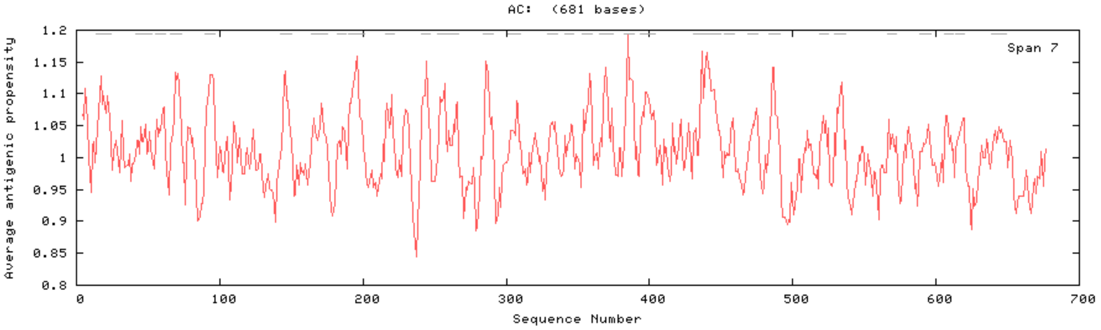

n	Position	Sequence	Length
1	14-24	DVASAPLAAYR	11
2	41-53	QNLSGLSRAFSAK	13
3	55-62	AGSDVIGI	8
4	66-73	TTNSCVAV	8
5	90-97	TTPSVVAF	8
6	142-150	KMVPYKIVR	9
7	164-175	QYSPSQIGAFIL	12
8	182-188	AEAYLGK	7
9	190-201	ISKAVITVPAYF	12
10	216-222	AGLDVQR	7
11	241-247	GLIAVFD	7
12	252-267	TFDVSILEISNGVFEV	16
13	284-291	TLLEYLVN	8
14	301-310	LSKDKLALQR	10
15	329-336	DINLPFIT	8
16	341-347	GAKHLNI	7
17	353-360	KFESLVNH	8
18	365-375	TRIPCKNCLKD	11
19	382-389	EVDEVLLV	8
20	394-404	RVPRVQEIVSE	11
21	431-450	LRGDVKELLLLLDVTPLSLGI	20
22	452-459	TLGGIFTR	8
23	469-476	TKKSQVFS	8
24	483-491	TQVGIKVLQ	9
25	519-525	PQIEVTF	7
26	529-537	ANGIVTVSA	9
27	566-572	KEAELFA	7
28	589-596	DTTIYSIE	8
29	606-612	IPSEVAK	7
30	614-620	IEDAVAD	7
31	639-649	AANKAVSKIGE	11

Fig. S1

Potri.006G116800.1
(Spot no.44)

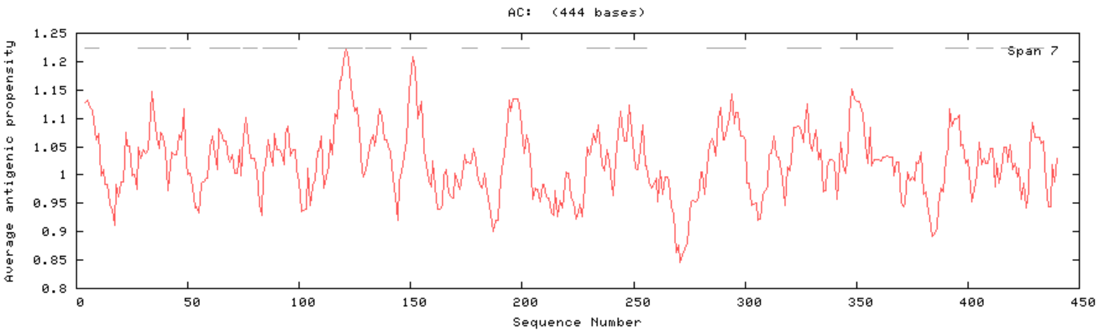

n	Position	Sequence	Length
1	4-10	TIVSVKA	7
2	28-40	TSDGVLSRAAVPS	13
3	42-51	ASTGVYEALE	10
4	60-73	LGKGVSKAVGNVNT	14
5	75-81	IGPALIG	7
6	84-99	PTEQVAIDNLMVQQLD	16
7	113-128	GANAILAVSLAVCKAG	16
8	130-141	HAKGIPLYKHIA	12
9	146-157	NKNLVLPVPAFN	12
10	173-180	EFMILPTG	8
11	191-203	GAEVYHHLKSVIK	13
12	229-239	GLELLKTAIAK	11
13	242-256	YTGKVVIGMDVAASE	15
14	283-300	ALKDLYKSFVSEYPIVSI	18
15	319-334	IGEKVQIVGDDLLVTN	16
16	343-366	KEKACNALLLKVNQIGSVTESIEA	24
17	390-400	FIADLSVGLAT	11
18	404-411	KTGAPCRS	8
19	415-421	AKYNQIL	7
20	428-434	GAEAVYA	7

Fig. S1

Potri.015G131100.3 (Spot no.51)

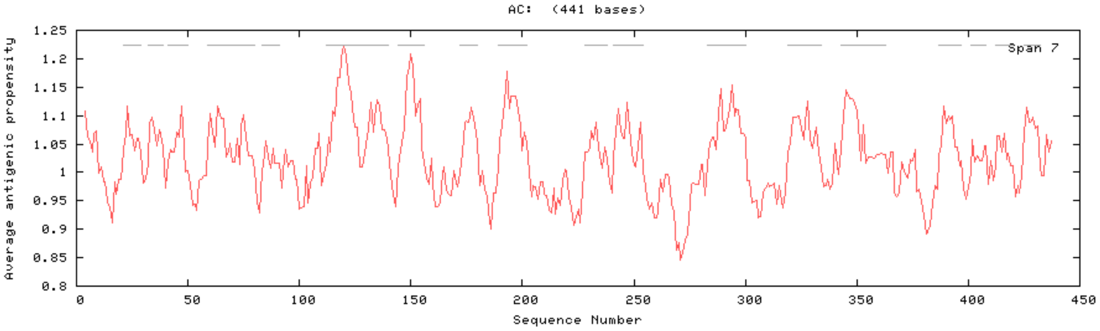

n	Position	Sequence	Length
1	21-29	VEADILLSD	9
2	32-39	YARAAVPS	8
3	41-50	ASTGVYEALE	10
4	59-80	LGKGVLKAVGNVNSIIGPALIG	22
5	83-91	PTEQVQIDN	9
6	112-140	GANAILAVSLAVCKAGAMVKKIPLYQHIA	29
7	144-156	GNKTLVLPVPAFN	13
8	172-180	EFMILPVGA	9
9	189-202	MGVEVYHHLKSVIK	14
10	228-238	GLELLKTAIAK	11
11	241-254	YTGKVVIGMDVAAS	14
12	283-300	SLKNVYKSFVADYPIVSI	18
13	319-334	VGEQVQIVGDDLLVTN	16
14	343-363	SCNALLLKVNQIGSVTESIEA	21
15	387-397	FIADLSVGLST	11
16	401-408	KTGAPCRS	8
17	412-420	AKYNQLLRI	9
18	425-432	GSAAVYAG	8

Fig. S1

Potri.019G067200.1 (Spot no.66)

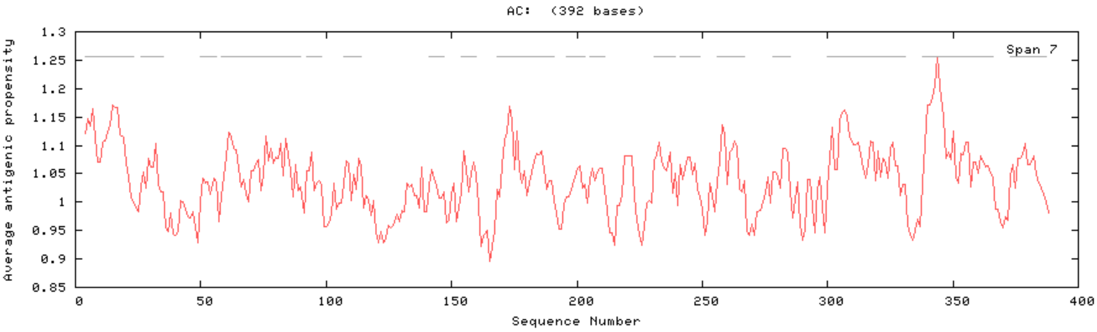

n	Position	Sequence	Length
1	4-23	KVVSSAIISFSLFLLLASTA	20
2	26-35	QSKGVFDVTK	10
3	50-56	WKDACAS	7
4	58-90	NPSKVLIPSGTYSLRKLTLAGPCKAAIELQVDG	33
5	92-98	LKAPVDP	7
6	107-114	VNFGHVDK	8
7	141-147	DCDSLPM	7
8	154-160	ITNALVR	7
9	168-191	KNFHVNVLGCKNLTFQHFTVSAPG	24
10	196-203	TDGIHVGQ	8
11	205-211	TGIYIID	7
12	231-239	HITGVTCGP	9
13	241-249	HGISVGSLG	9
14	256-267	PVSGIFVKNCTL	12
15	278-285	SWPALYGG	8
16	300-331	VQNPVIIDQVYCPWNQCSLKAPSKVKISGVSF	32
17	338-366	SATPVVVQIACSSGFPCEKVELADIKLAY	29
18	373-387	SKSQCSNVKPIISGI	15

Fig. S1

Potri.002G034400.1 (Spot no.78)

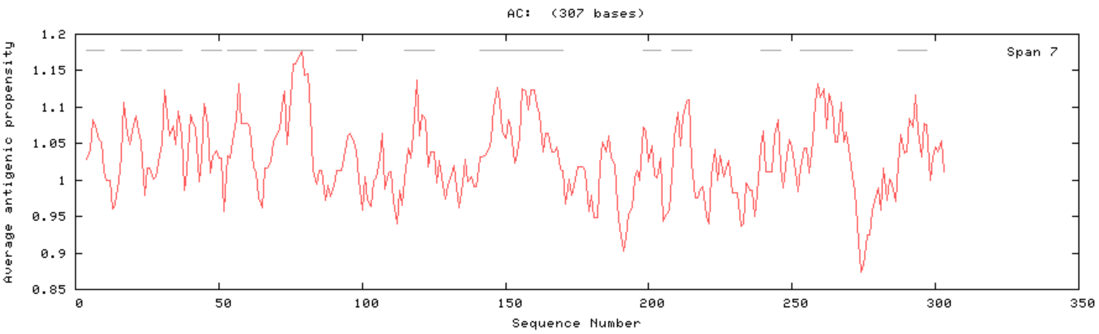

n	Position	Sequence	Length
1	4-10	KSKILII	7
2	16-23	IGKFIVEA	8
3	25-37	AKAGHPTFALVRE	13
4	44-51	VKRELVEK	8
5	53-63	KNLGVTLIHGD	11
6	66-83	GHDNLVKAIKQVDVVISA	18
7	91-98	DQTKIIAA	8
8	115-125	MDVDHVNAVEP	11
9	141-170	EAAGIPYTYVPSNFFAAYYLPTLAQFGLTA	30
10	198-204	TIKAVDD	7
11	208-215	LNKTVLIK	8
12	239-246	EKTFVPEE	8
13	253-271	QESPIPINIVLSINHSALV	19
14	287-297	EASELYPDVKY	11

Fig. S1

Potri.012G114900.1 (Spot no.80)

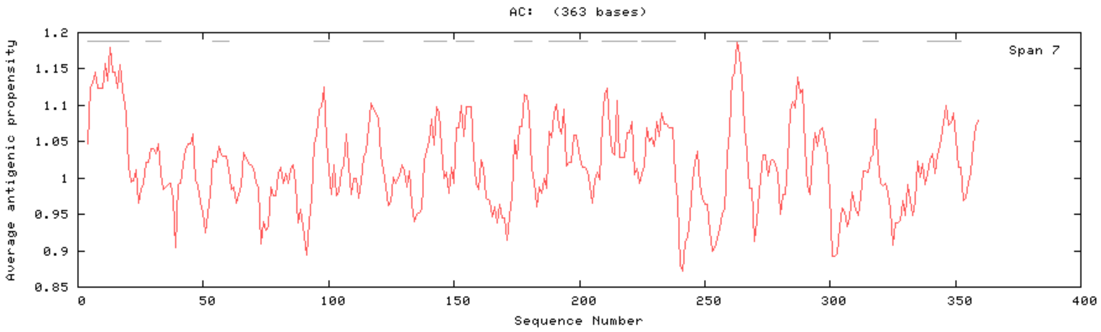

n	Position	Sequence	Length
1	4-20	IAAIQCALTAILLVSTT	17
2	27-33	SPIPADP	7
3	54-60	IDPALEA	7
4	94-100	ERVIVDI	7
5	114-122	SKPFVTFLG	9
6	138-147	EYGTVYSATL	10
7	151-158	ADYFVAAN	8
8	174-181	GEQAVALR	8
9	188-203	AFYNCRLIGFQDTLCD	16
10	209-223	LFKDCYIEGTVDYIF	15
11	225-238	SGKSLYLGTELHVI	14
12	259-267	GFSFVHCKV	9
13	273-279	KGAYLGR	7
14	283-290	ARPRVVFS	8
15	293-299	TMSSVVN	7
16	313-319	QTALFGE	7
17	339-352	LTPDQAAPFISLGF	14

Fig. S1

Potri.010G117900.1 (Spot no.84)

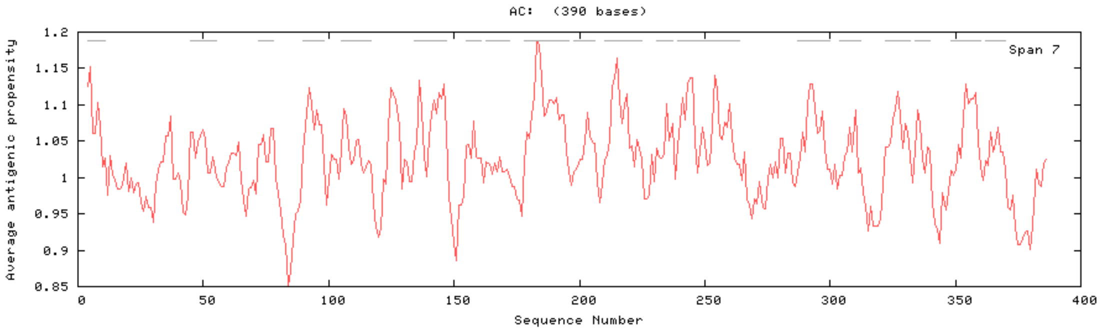

n	Position	Sequence	Length
1	4-11	ANLVKLNA	8
2	45-55	TDELVQTAKTI	11
3	72-78	CGKRLAS	7
4	90-98	AYRQLLLTT	9
5	105-117	ISGAILFEETLYQ	13
6	134-147	IVPGIKVDKGLVPL	14
7	155-161	WCQGLDG	7
8	163-172	ASRSAEYYKQ	10
9	178-196	KWRTVVSIPCGPSALAVKE	19
10	198-206	AWGLARYAA	9
11	210-225	DNGLVPIVEPEILLDG	16
12	231-237	RTLEVAE	7
13	239-264	VWSEVFYYLAENNVVFEGILLKPSMV	26
14	287-300	LKRRVPPAVPGIMF	14
15	304-312	GQSEVQATL	9
16	322-332	NPWHVSFSYAR	11
17	334-340	LQNTVLK	7
18	348-360	NVEAAQKSLLVRA	13
19	362-370	ANSLAQLGR	9

Fig. S1

Potri.009G018600.1 (Spot no.95)

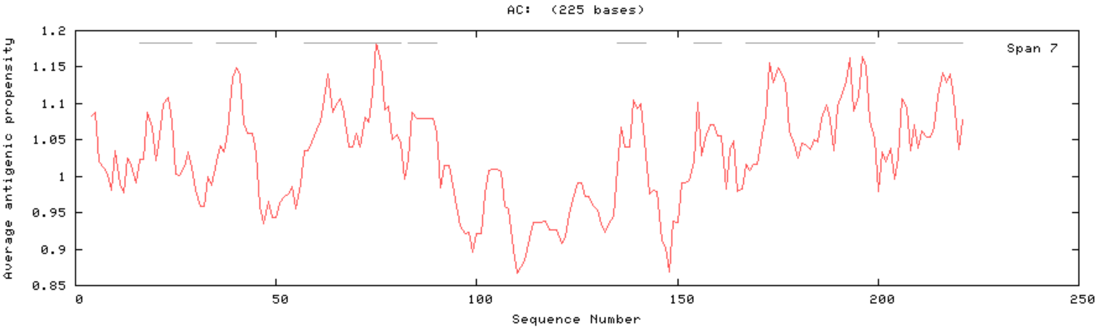

n	Position	Sequence	Length
1	16-29	SLNEFLVGKSYISG	14
2	35-45	DDIKVYGAVLE	11
3	57-81	WYESVSSQLALSFPGKAVGVSICGK	25
4	83-90	AAAAPVEA	8
5	135-142	GKSSVLMD	8
6	154-161	ELEKAVRS	8
7	167-199	LFWGASKLVPVGYGIKKLQIMLTIVDDLVSVDS	33
8	205-221	LTVEPCNEYIQSCDIVA	17

Fig. S1

Potri.001G034400.1 (Spot no.117)

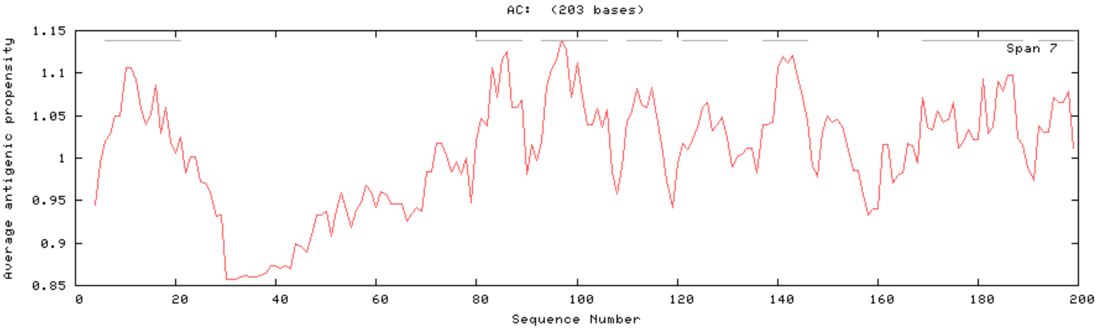

n	Position	Sequence	Length
1	6-21	QEELLAAQLEIQKILE	16
2	80-89	TIPGVSRVTV	10
3	93-106	KNILFVISKPDVFK	14
4	110-117	SDTYVIFG	8
5	121-130	IEDLSSQLQT	10
6	137-146	KAPDLSHVIS	10
7	169-189	DIELVMTQAGVSRSKAVKALK	21
8	192-199	DGDIVSAI	8

Fig. S1

Potri.008G056300.2 (Spot no.133)

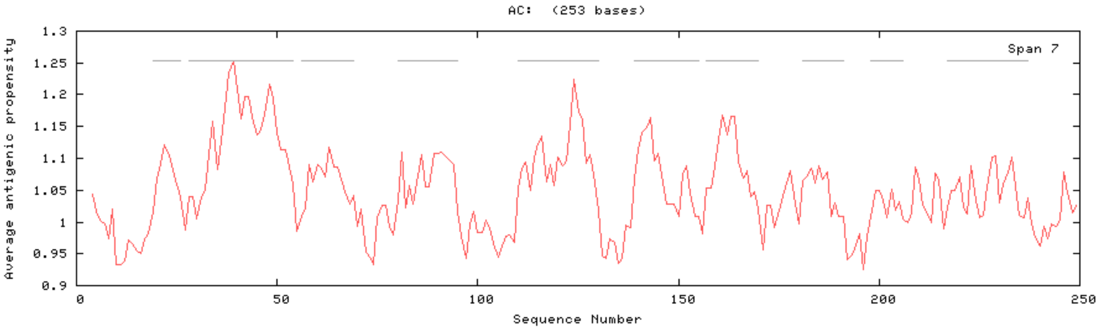

n	Position	Sequence	Length
1	19-26	EVKKIVSA	8
2	28-54	NNSQVPSSDVVEVVVSPPFVFLPLVKS	27
3	56-69	LRPDFHVAAQNCWV	14
4	80-95	SAEMLVNLDIPWVILG	16
5	110-130	VGDKVAYALSQGLKVIACVGE	21
6	139-155	STVEVVAAQTKAIAARV	17
7	157-170	NWADVVLAYEPVWA	14
8	181-191	QAQEVHYELRK	11
9	198-206	SPEVAATTR	9
10	217-237	NCKELAAKPDVDGFLVGGASL	21

Fig. S1

Potri.013G092600.1 (Spot no.135)

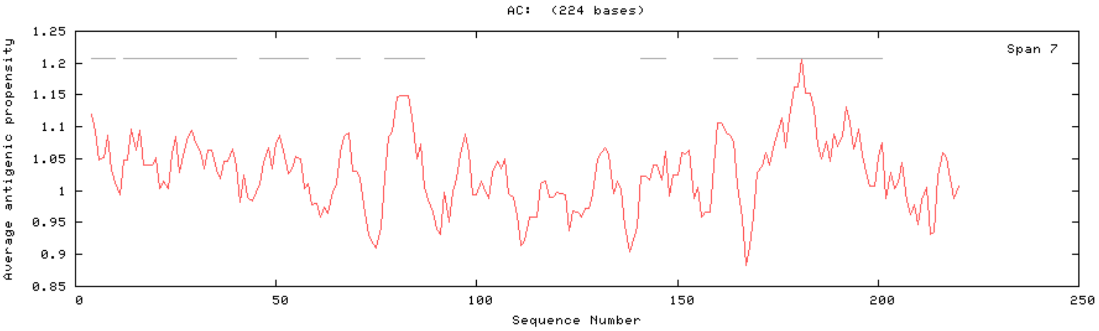

n	Position	Sequence	Length
1	4-10	RSLVSRK	7
2	12-40	LGLGLKLQFRGLQTFSLPDLPYDYGALEP	29
3	46-58	IMQLHHQKHHQTY	13
4	65-71	SLEQLHH	7
5	77-87	DSSAVVKLQSA	11
6	141-147	EGAAVQG	7
7	159-165	SKKLVVE	7
8	170-201	QDPLVTKGPLVPLLGVDVWEHAYYLQYKNVRP	32

Fig. S1

Potri.010G195700.1
(Spot no.142)

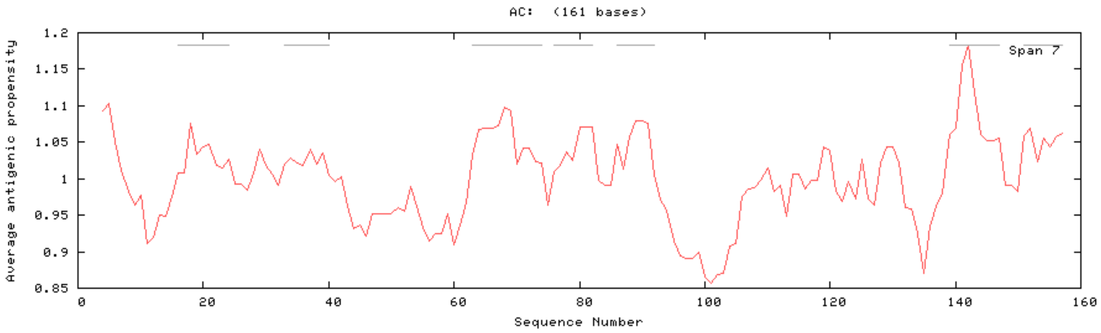

n	Position	Sequence	Length
1	16-24	IFDPFSLDI	9
2	33-40	FTSTAISA	8
3	63-74	PEAHVFKADLPG	12
4	76-82	KKEEVKV	7
5	86-92	EGRVLQI	7
6	139-147	GVLTVTVPK	9
7	151-157	KKPDVKA	7

Fig. S1

Potri.001G254700.1 (Spot no.144)

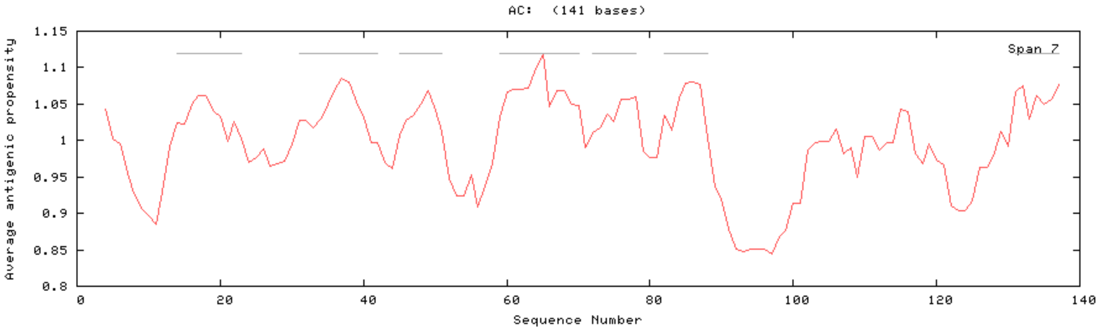

n	Position	Sequence	Length
1	14-23	SSIIFDPFSS	10
2	31-42	KDFPFPSSSLIP	12
3	45-51	NSAFVST	7
4	59-70	PEAHVFKADLLG	12
5	72-78	KKEEVKV	7
6	82-88	DDRVLQI	7
7	131-137	KKHDVKA	7

Fig. S1

Potri.009G147900.1 (Spot no.156)

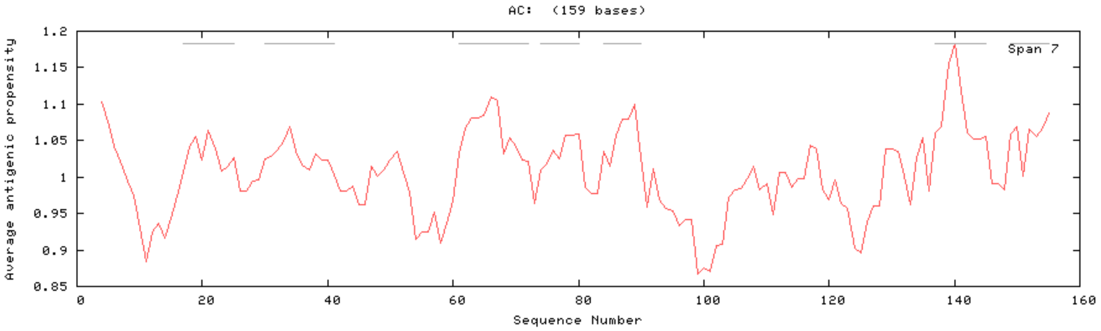

n	Position	Sequence	Length
1	17-25	FDPFSSLDI	9
2	30-41	KDLTPFISSSNS	12
3	61-72	PEAHVFQADLPG	12
4	74-80	KKEEVKV	7
5	84-90	DDRVLQI	7
6	137-145	GVLTVTVPK	9
7	149-155	KKPDVKT	7

Fig. S1

Potri.018G083500.1 (Spot no.158)

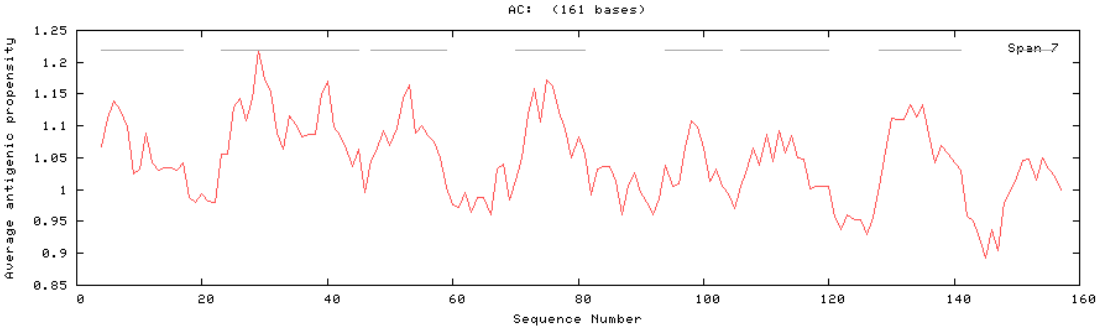

n	Position	Sequence	Length
1	4-17	IAVGDVLPDGKLAY	14
2	23-45	QLQEVSVHSLVAGKKVILFGVPG	23
3	47-59	FTPTCSLKHVPGF	13
4	70-81	GVTEILCISVND	12
5	94-103	ENKHVKFLAD	10
6	106-120	ATYTHALGLELDLQE	15
7	128-141	RRFALLVDDLKVKA	14
8	150-156	FTVSSAD	7

Fig. S1

Potri.001G392400.1 (Spot no.161)

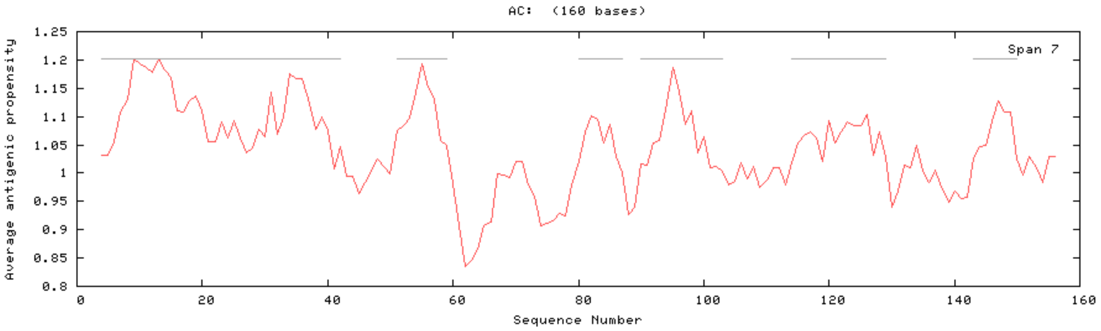

n	Position	Sequence	Length
1	4-42	SFTIAAFVLCFSSLLISAYAADYLDVEGKVYCDPCRVEF	39
2	51-59	PAAKVKLVC	9
3	80-87	TYRLPVAG	8
4	90-103	EDDICEVRLVESSR	14
5	114-129	DSARILLTKNVGVVDK	16
6	143-150	AQPECADV	8

Fig. S1

Potri.011G111300.1 (Spot no.164)

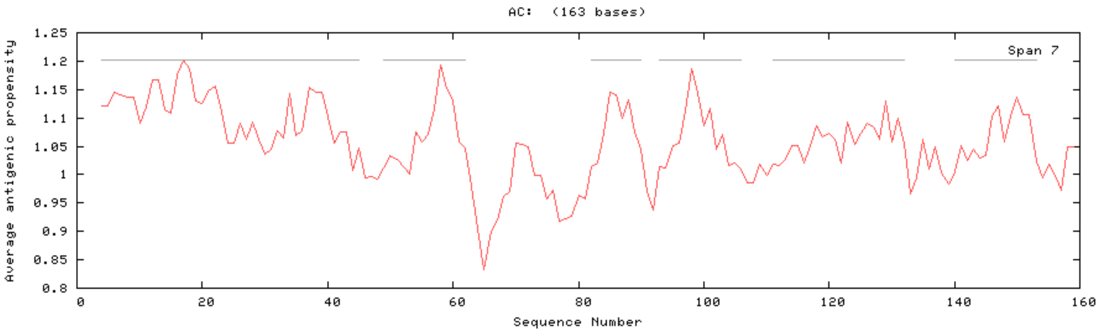

n	Position	Sequence	Length
1	4-45	CFTVAAFVATALCFSSVLISAYAADYLDVEGKVYCDTC	42
2	49-62	ISDAIPGAKVKLVC	14
3	82-90	GTYRLPVVG	9
4	93-106	EEDICEVRLVESPR	14
5	111-132	EPFKSVDSARILLTKNVGVVDN	22
6	140-153	GYMKKVAQPECAKV	14

Fig. S1

Potri.007G018000.1 (Spot no.181)

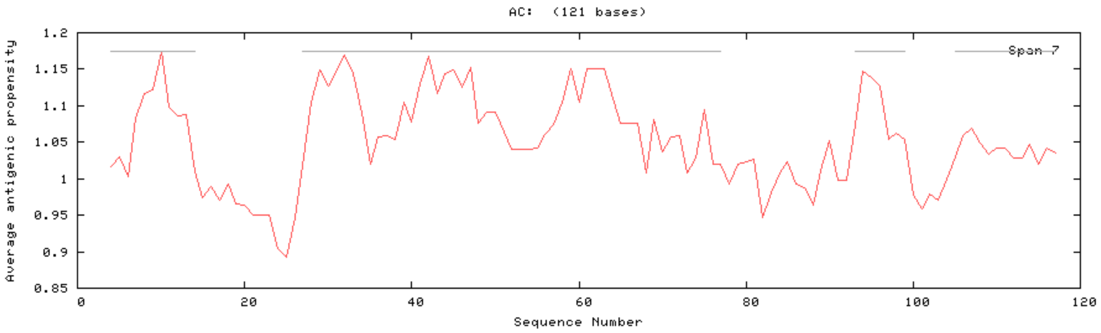

n	Position	Sequence	Length
1	4-14	EDGQVIGCHTV	11
2	27-77	SKKLVVIDFAASWCGPCRVIAPFLAELARKLPDVIFLKV DVDELKTVAQDW	51
3	93-99	IVDKVVG	7
4	105-117	LQQAIAKHTAPAA	13

Fig. S1

Potri.006G093500.1 (Spot no.184)

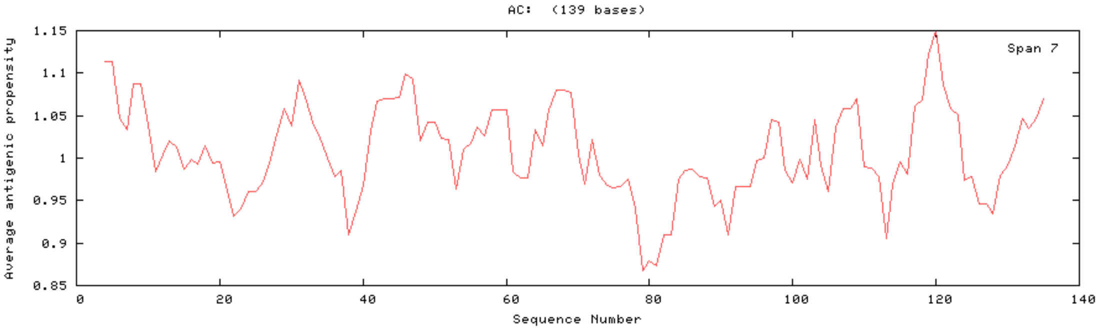

n	Position	Sequence	Length
1	4-10	IRSLLSN	7
2	28-35	SSFASAQV	8
3	41-52	PEAHVFKADLPG	12
4	54-60	KKEEVKV	7
5	64-70	EGRVLQI	7
6	117-123	GVLTVTI	7

Fig. S1

Potri.009G146200.1
(Spot no.197)

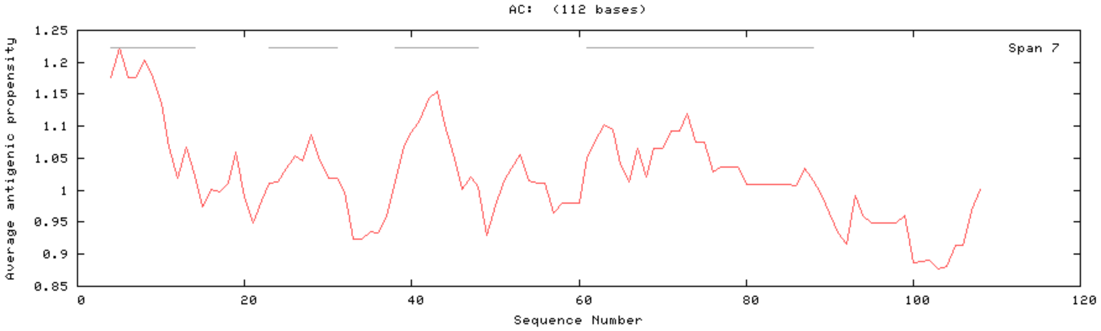

n	Position	Sequence	Length
1	4-14	VAAYLLAVLGG	11
2	23-31	LKNILGSVG	9
3	38-48	RIELLLSSVKG	11
4	61-88	KLASVPSGGGVAVSAGAAPAAAGGAAPA	28

Fig. S1

Potri.003G047700.1 (Spot no.200)

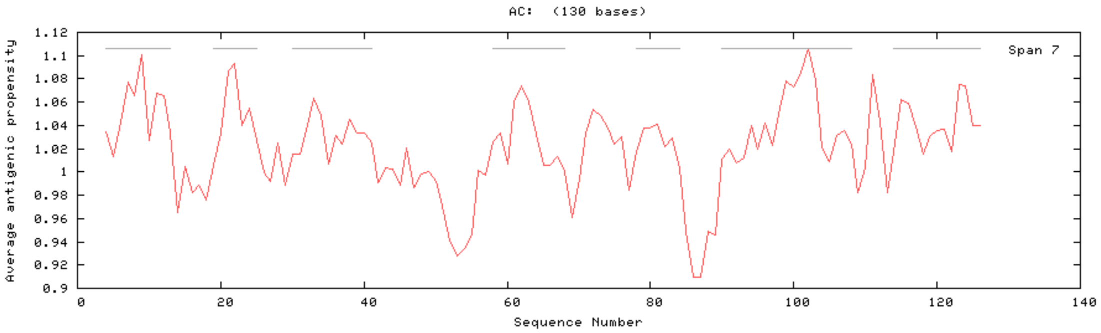

n	Position	Sequence	Length
1	4-13	QTYVDDHLMC	10
2	19-25	HLTAAAI	7
3	30-41	GSVWAQSATFPQ	12
4	58-68	GSLAPTGLHIG	11
5	78-84	EPGAVIR	7
6	90-108	GGITVKKTAQALIFGIYDE	19
7	114-126	QCNMIVERLGDYL	13

Fig. S1

Potri.006G235200.2 (Spot no.201)

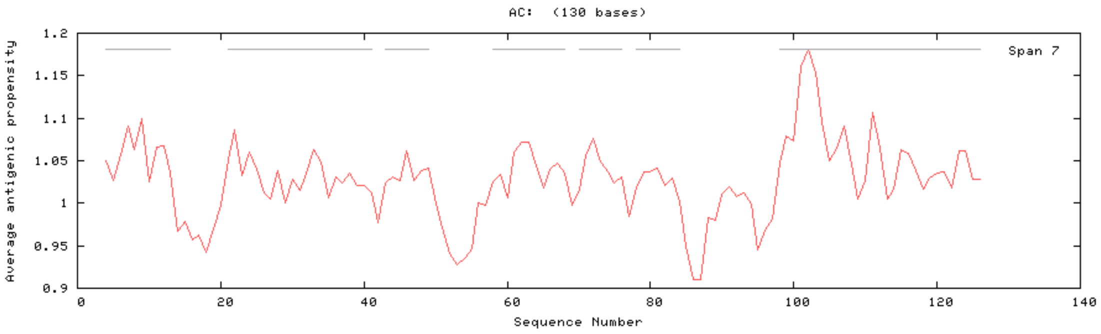

n	Position	Sequence	Length
1	4-13	QQYVDEHLMC	10
2	21-41	TSAAIIGHDGSVWAQSATFPQ	21
3	43-49	TAEEVAA	7
4	58-68	GSLAPTGLFLG	11
5	70-76	AKYMVIQ	7
6	78-84	EAGAVIR	7
7	98-126	NQALVIGVYDEPLAPGQCNMIVERLGDYL	29

Fig. S1

Potri.001G190800.2 (Spot no.203)

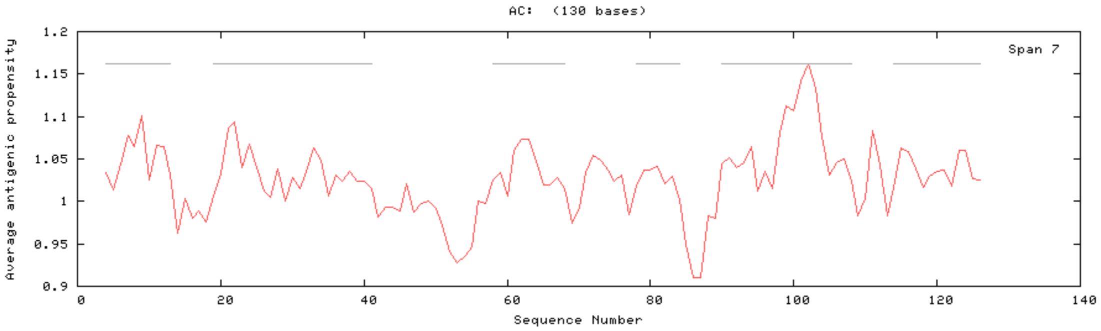

n	Position	Sequence	Length
1	4-13	QTYVDDHLMC	10
2	19-41	HLTAAAIIGHDGSVWAQSATFPQ	23
3	58-68	GSLAPTGLHLG	11
4	78-84	EPGAVIR	7
5	90-108	GGVTVKKTSQALVIGLYDE	19
6	114-126	QCNMIVERLGDYL	13

Fig. S1

Potri.005G232700.2 (Spot no.208)

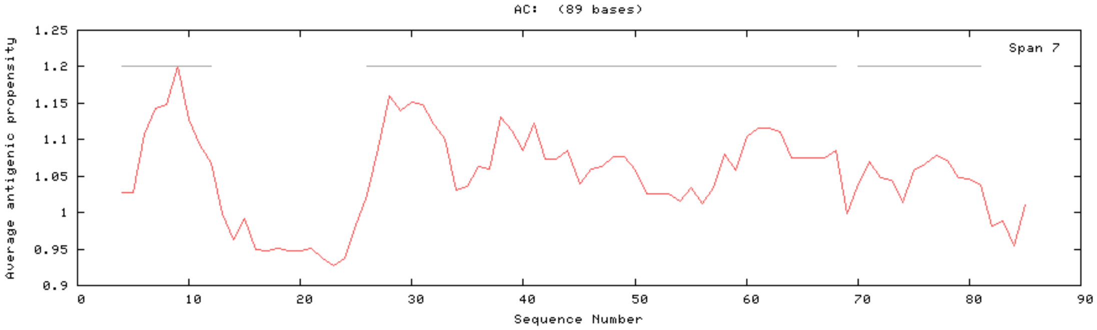

n	Position	Sequence	Length
1	4-12	EGQVIACHT	9
2	26-68	SQKLIVVDFTASWCPPCKMIAPIFAELAKKFPNVTFLKVDVDE	43
3	70-81	KVKNYAFTYLML	12

Fig. S1

Potri.018G057600.7 (Spot no.216)

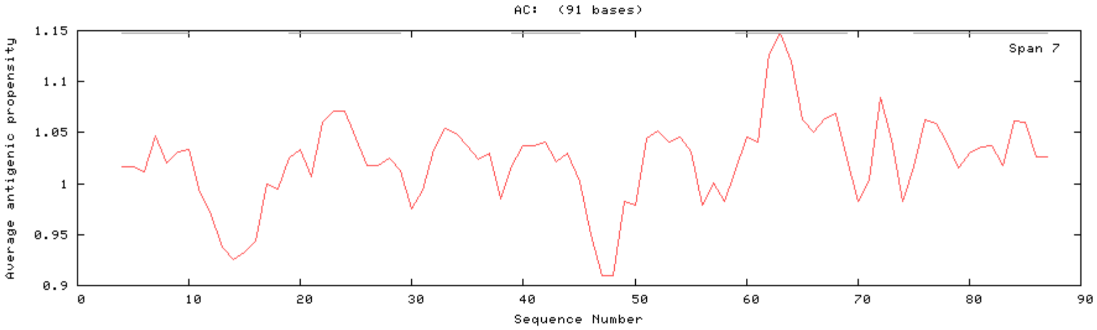

n	Position	Sequence	Length
1	4-10	TQEEVSA	7
2	19-29	GSLAPTGLFLG	11
3	39-45	EPGAVIR	7
4	59-69	NQALIIGVYDE	11
5	75-87	QCNMIVERLGDYL	13

Fig. S1
