# Supplementary figures and images for: Proteomic analysis and candidate allergenic proteins in Populus deltoides CL. “2KEN8” mature pollen
Source: Front Plant Sci. 2015 Jul 29;6:548. doi: 10.3389/fpls.2015.00548 (PMC4518142; doi:10.3389/fpls.2015.00548)

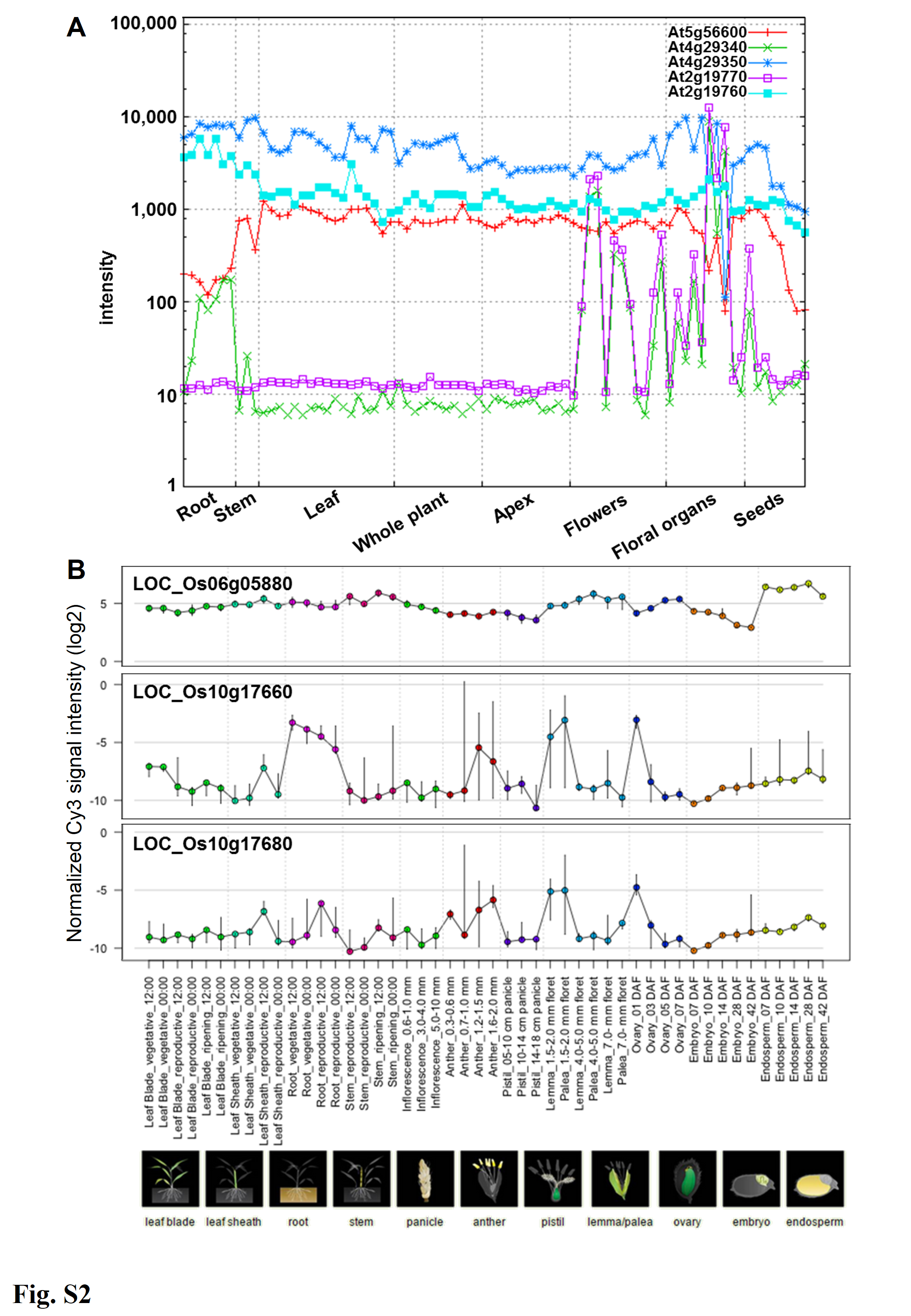

Supplement: Figure S2 — Expression patterns of Profilin genes in various tissues of Arabidopsis (A) and rice (B). The microarray data was obtained from AtGenExpress Visualization Tool (AVT, http://jsp.weigelworld.org/expviz/) for Arabidopsis and the Rice Expression Profile Database (RiceXPro, http://ricexpro.dna.affrc.go.jp/) for rice. [file Image2.TIF]
