# Supplementary material for: Proteomic analysis and candidate allergenic proteins in Populus deltoides CL. “2KEN8” mature pollen
Source: Front Plant Sci. 2015 Jul 29;6:548. doi: 10.3389/fpls.2015.00548 (PMC4518142; doi:10.3389/fpls.2015.00548)
Supplement: Table S1 — Sequences of qRT-PCR primers. [file Table1.DOCX]

**Table S1. The sequences of qRT-PCR primers.**

| **Spot no.** | **Locus name** | **Forward Primer** | **Reverse Primer** |
| --- | --- | --- | --- |
| 5 | Potri.001G087500 | CGAAAGAGGAGGCAACAAAG | CAATCAACCTCTCGCTGTCA |
| 22 | Potri.009G079700 | ACCAGACGCAGGTTGGTATC | CCTTGGCAGAGACAGTGACA |
| 44 | Potri.006G116800 | TCATCAACGGTGGATCACAT | CCAACATTTGTTGCATCCTG |
| 66 | Potri.019G067200 | GTGACAGCCTCCCAATGAAT | GCCTGTGCCAATTTTTGAAT |
| 78 | Potri.002G034400 | ATTGGGAGCATGCAAATAGC | GCAGCAAAGAAGTTGGAAGG |
| 80 | Potri.012G114900 | CGCCTAGTAACAAGCCAACC | CTTGTCCCCAGAAATCCTCA |
| 135 | Potri.013G092600 | GTCCCTCGAGCAACTTCATC | AGCACCCTCTGTGCTCATTT |
| 158 | Potri.018G083500 | TTGCCGGTAAAAAGGTCATC | CTTCATCACAAAGGGGTCGT |
| 161 | Potri.001G392400 | CCAAAATCAGCGAGGGAATA | AGAATCAATGCTCCGGAATG |
| 164 | Potri.011G111300 | TGCTTCTCCTCCGTTCTCAT | GTCAATGTGCCATTCTCACG |
| 181 | Potri.007G018000 | TTGATTTTGCTGCTTCATGG | TTCCTTGCTCCCACAACTTT |
| 197 | Potri.009G146200 | GATCGAGTTGCTGTTGTCCA | CAGGCTGAAGCCCATATCAT |
| 200 | Potri.003G047700 | TGGATCTCTAGCCCCAACTG | CTGGAGTCAATGGCTCATCA |
| 201 | Potri.006G235200 | GGTGGCGCTAAGTACATGGT | CCAAGCCTCTCCACAATCAT |
| 203 | Potri.001G190800 | ACATGGTGATCCAGGGAGAG | GAGCACCAGCATTACCCTTC |
| 216 | Potri.018G057600 | GTGGCAGGTGTACGTTGATG | AAGGAACAACCCAGTTGGTG |
| *Actin* |  | GTGCTTCTAAGTTCCGAACAGTGC | GACTACCAAAGTGTCTGACCACCA |
